# Modeling Frailty Risk Based on Biochemical and Hematological Parameters Using Multiple and Logistic Regression: Cross-Sectional Analysis of the Toledo Study for Healthy Aging

**DOI:** 10.2196/76500

**Published:** 2026-10-30

**Authors:** Francisco Zamudio, Diego Arauna, Francisco Garcia, Cecilia Albala, Victoria Villalobos, Eduardo Fuentes, Ivan Palomo

**Affiliations:** 1 Department of Public Health, University of Talca, Talca, Chile; 2 Thrombosis Research Center, Interuniversity Center for Healthy Aging, VITALIS Longevity Center, Medical Technology School, Department of Clinical Biochemistry and Immunohaematology, Faculty of Health Sciences, University of Talca, Avenida Lircay S/N, Talca, 3460000, Chile, 56 712200200; 3 Department of Geriatrics, Hospital Virgen del Valle, Complejo Hospitalario Universitario de Toledo, Toledo, Spain; 4 Instituto de Nutrición y Tecnología de los Alimentos, University of Chile, Santiago, Chile

**Keywords:** aging, frailty, frailty risk prediction, hematological parameters, biochemical parameters, logistic regression analysis

## Abstract

**Background:**

Frailty is a complex syndrome characterized by diminished physiological reserves, resulting in increased vulnerability among older adults. Scalable tools that leverage routinely collected laboratory data could enable earlier risk stratification in aging populations.

**Objective:**

The aim of the study is to model frailty risk using Frailty Trait Scale (FTS)-12 scores and biochemical or hematological parameters, and to examine sex-specific patterns in predictors and performance.

**Methods:**

This study analyzed data from a population-based cohort of 1485 individuals aged 65 years or older, drawn from the Toledo Study for Healthy Aging (TSHA). Multiple and logistic regression analyses were used to investigate the relationships between biochemical and hematological parameters, and the FTS-12 score. Predictive models were developed separately for female and male participants, incorporating both single and composite risk factors. Model performance was evaluated using correlation coefficients, coefficients of determination, Akaike information criterion, log-likelihood values, and receiver operating characteristic curves, which summarize how well the models distinguish between higher and lower frailty risk.

**Results:**

The regression models accounted for approximately 40% of the variability in FTS-12 scores, revealing distinct risk factors for each sex. Gamma-glutamyl transferase, albumin, and age were kept as key predictors in the selected models. Several composite risk factor functions were also significantly associated with the FTS-12 score. Correlations between observed and predicted FTS-12 scores were moderately strong, with values of 0.621 (*P*<.001) for female participants and 0.615 (*P*<.001) for male participants. Logistic regression models showed moderate-to-good discriminative performance for high frailty probability, achieving receiver operating characteristic area under the curve values of 0.799 for female participants and 0.824 for male participants.

**Conclusions:**

This analysis is an exploratory proof-of-concept study, and the developed models do not constitute validated clinical prediction tools. Routine laboratory profiles demonstrate moderate, sex-specific predictive performance for FTS-12–defined frailty and may, in principle, complement comprehensive geriatric assessment by facilitating the early identification of patients who could benefit from further evaluation and closer clinical follow-up. External validation in independent cohorts is required before any clinical use, alongside longitudinal designs with repeated biomarker measures and integration of additional biological layers, to enhance generalizability and confirm clinical utility. Within these limits, the analysis highlights the potential of routine laboratory data to support digitally enabled frailty screening and workflow-integrated risk stratification in primary care and remote geriatric care settings.

## Introduction

Frailty is a multifaceted clinical syndrome characterized by diminished strength, endurance, and physiological function, leading to increased vulnerability to dependency and mortality [1-3]. Its complexity stems from several subtypes, including physical, cognitive, oral, and social frailty [4-6]. Physical frailty and physiological decline have been the most extensively studied. Fried et al [3] distinguished frailty as a condition separate from aging, comorbidity, and disability, noting its clinical reversibility and its role as a precursor to dependency in older adults. While unhealthy aging provides a context for frailty development, it is not an inevitable outcome, as individuals may exhibit varying levels of frailty over time [1,7-9]

Assessment tools such as the Fried Frailty Phenotype and the Frailty Index (FI) are routinely used in clinical practice to evaluate frailty. The Fried Frailty Phenotype identifies 5 dimensions—weakness, low endurance, slowness, low physical activity, and weight loss—and classifies individuals as frail if they perform poorly in 3 or more domains [3]. In contrast, the FI conceptualizes frailty as the accumulation of deficits that predispose individuals to adverse outcomes, including mortality and hospitalization [10]. Despite their widespread validation, both methods have limitations in clinical settings, such as being time-intensive and challenging for monitoring subtle changes over time [11,12]. To address these gaps, newer approaches have expanded the conceptualization of frailty. García-García et al [11,13] introduced the Frailty Trait Scale (FTS)-12, which considers frailty as a biological trait encompassing additional domains, including vascular and nervous system health and nutritional status. The FTS has 3 validated versions (FTS-12, FTS-5, and FTS-3), each capturing different dimensions of frailty. These scales exhibit high sensitivity and accuracy in detecting frailty and predicting adverse health outcomes, such as hospitalization, fractures, and mortality. Among these, FTS-12 is effective in comprehensively assessing frailty and its associated risks [11].

Since 2022, we have pursued a screening-oriented approach to investigate whether routinely available laboratory data can help identify older adults at increased risk of frailty. In this study, we examined whether common biochemical and hematological parameters could be used to predict the FTS-12 score and to identify individuals more likely to fall within the highest frailty range. Beyond its statistical relevance, this approach may contribute to health care innovation by evaluating whether low-cost, routinely available laboratory data can support practical frailty risk stratification within digitally enabled geriatric care. In this context, this study aims (1) to develop an equation to predict an FTS-12 score of an older individual based on their biochemical profile; (2) to determine whether frailty, as assessed by the FTS-12 scale, can be predicted to fall within the upper range of the distribution using routinely available blood-based biomarkers; and (3) to establish exploratory criteria for selecting predictive equations based on observed frailty estimates.

This study is conceived as an exploratory, proof-of-concept analysis, and the resulting models should not be regarded as validated clinical prediction tools. The approach is not intended to replace comprehensive clinical assessment of frailty; rather, it explores whether routinely available laboratory data carry meaningful frailty-related signals that could, pending external validation, eventually support preliminary screening of older individuals with a high probability of frailty.

## Methods

### Data Origin

This analysis used data from the Toledo Study for Healthy Aging (TSHA), a prospective, population-based cohort study investigating the determinants and consequences of frailty syndrome in older adults living in Toledo, Spain [14]. The study included 1972 participants who completed a home-based interview and physical evaluation, with biological samples provided by 1752 individuals for laboratory analysis. Baseline data were collected between June 2006 and September 2009, and participants were followed for an average of 3.5 (SD 1.19; range 0.30‐4.79) years to evaluate study outcomes [11].

### FTS-12, Blood Collection, and Measured Traits

The FTS-12 served as the dependent variable in statistical analyses. This scale includes 12 items across 7 dimensions. To calculate the FTS-12 score for each participant, the TSHA collected data in the following areas: (1) self-reported comorbidities, (2) basic and instrumental activities of daily living, (3) depressive symptoms, and (4) cognitive function. Trained nurses conducted physical examinations and clinical tests at participants’ homes to supplement these data. Detailed assessment methods are provided in García-García et al [11].

Participants also provided 12-hour fasting blood samples, from which two primary categories of variables were derived: (1) 15 variables assessing blood characteristics (hemogram) and (2) 19 variables measuring biochemical characteristics (biochemical profile), along with an added 12 unique biochemical traits not included in the standard profile. In total, 46 blood-related variables, combined with participants’ age, were included in the original database.

### Ethical Considerations

The TSHA was approved by the clinical research ethics committee of the Complejo Hospitalario de Toledo, Toledo, Spain. The original cohort study (“Estudio Toledo de Envejecimiento”) received ethics approval on March 30, 2005 (application 22). In addition, the specific research project entitled “Predictors of frailty, disability, cognitive impairment, vascular events, hospitalization, and mortality in a cohort of adults older than 64 years. Toledo Study for Healthy Aging” was approved on July 14, 2010 (application 93). All participants provided written informed consent before enrollment. This study is a secondary analysis of previously collected cohort data.

### Statistical Analysis

#### Exploratory Data Analysis

The software programs JMP (version 13.2) and SAS Enterprise Guide (version 7.1; SAS Institute Inc) were used to perform descriptive statistical methods for each sex independently and to identify potential data anomalies [15,16]. Descriptive statistics included (1) examining data dispersion for individual variables and correlations between potential explanatory variables, (2) identifying plausible probability distributions for each variable, (3) detecting potential outliers mainly by using box plot analysis, and (4) analyzing the relationship between the main dependent variable (FTS-12) and possible explanatory variables. The final database included measurements from 1485 individuals aged 65 years and older, comprising 848 female participants and 637 male participants.

#### Missing Data

Missing data were present in less than 5% of observations across study variables. Analyses were conducted using complete cases only (listwise deletion). This approach is valid under the reasonable assumption that data were missing completely at random. Since the small amount of missingness was mostly caused by small problems during blood sample collection, this assumption was deemed reasonable. Given the low proportion of missingness, potential bias due to exclusion of incomplete cases was considered negligible. The exclusion of missing data is the default procedure in SAS Enterprise Guide (version 7.1) software.

#### Modeling Regression Equations to Predict the FTS-12 Score

Two predictive models of FTS-12 scores were developed based on the following criteria: (1) sex and (2) available biological information, which included data from the biochemical profile and hemogram. The models were constructed using the theory of linear models in the framework of multiple regression analysis [17-19]. The mathematical formulations of the regression models are provided in Multimedia Appendix 1. Additional details regarding the model and its underlying assumptions are provided in Multimedia Appendix 2. We used a 2-stage, data-driven selection process. Predictors were initially screened based on their Pearson correlation with the outcome *r*≥0.18. A stepwise multiple regression was then applied to the remaining variables [17,18]. This approach was used for exploratory purposes, and the results should be interpreted cautiously. This approach was selected and justified because (1) we have a very large number of predictors (n=46); (2) this is a proof-of-concept analysis for predictive purposes (FTS-12 score and high-frailty probability, as described below), rather than an inferential analysis; (3) results must be treated as “exploratory and preliminary,” and the stepwise was used as a diagnostic tool, not a decision rule; and (4) the final model will be validated on independent data. Selection criteria included the coefficient of determination, residual mean square, Mallows *C*_p_ statistic, and Akaike information criterion (AIC). All statistical calculations were performed using the PROC REG procedure in SAS (version 9.4).

Bootstrapping in multiple regression can be used to support statistical inference when classical assumptions are questionable or when sample size is limited. Bootstrapping was unnecessary in our multiple regression analysis. The size of the database is large; residual effects followed a normal distribution, and there were no heteroscedasticity problems. Here, bootstrapping is optional. However, we considered performing bootstrapping as a complement to the selection process. We used the stepwise selection procedure, and bootstrapping was applied to construct empirical CIs for the parameter estimates. The “bootstrapping” analysis was then conducted for the selected multiple regression models using the PROC SURVEYSELECT and PROC REG procedures in SAS (version 9.4). We generated 1000 subsamples of size 500 from the dataset and fitted a regression model for each subsample. From these models, 95% CIs for each parameter and the model’s coefficient of determination were computed by calculating the 2.5% and 97.5% percentiles. A summary of bootstrapping is shown in Multimedia Appendix 2. Because the multiple regression model predicts a continuous outcome, calibration in the probabilistic sense was not applicable; instead, model performance was evaluated using goodness-of-fit measures (eg, *R*^2^) and residual diagnostics and agreement between observed and predicted values. These approaches provide an assessment of model performance analogous to calibration in probabilistic models.

#### Modeling the Odds of Reaching Maximum FTS-12 Scores

FTS-12 scores ranged from 1 to 100. To analyze frailty levels, the 3 quartiles of the distribution were calculated, dividing frailty data into 4 distinct quarters [11]. Each individual’s FTS-12 score was assigned to one of these 4 quarters, treating an individual’s FTS-12 value in any quarter as a binomial (binary) variable. The fourth quarter—standing for the highest frailty levels—was of primary interest in this study. This quarter included FTS-12 values above the third quartile (scores >55), representing the highest frailty scores in this cohort. This upper-quartile definition was used as a cohort-specific analytical threshold to identify the subgroup with the highest frailty burden within the present sample and should not be interpreted as a universal clinical cutoff for the FTS-12 scale. For any individual in the original TSHA data with an FTS-12 value equal to or above 55, a “1” was assigned under a newly created “fourth quarter” column, while scores below this threshold were assigned a “0.” We defined a binary response variable, *Y*, such that *Y*=1 if an individual’s FTS-12 score exceeded the third quartile (ie, fell within the highest—fourth—quarter), and *Y*=0 otherwise. Therefore, a multiple logistic regression analysis was conducted for the binary variable representing the fourth quarter, following the approach described by Hosmer et al [20]. Logistic regression enables the prediction of the odds of a particular event occurring as a function of observed values for a set of potential explanatory variables. Depending on the context, these variables are referred to as independent variables, risk factors, predictors, or regressors. Additional details regarding the logistic model and its underlying assumptions are provided in Multimedia Appendix 2. Regression analysis was conducted using JMP (version 13.2) software, along with the LOGISTIC procedure in SAS (version 9.4) and SAS Enterprise Guide (version 7.1). A stepwise regression approach, as outlined by Hosmer and Lemeshow [20], was applied as the selection method. Selection criteria included the coefficient of determination, residual mean square, Mallows *C*_p_ statistic, and AIC. Finally, we generated receiver operating characteristic (ROC) curves to illustrate how well the binary models distinguished between higher and lower frailty probability. As in the multiple regression analysis, this approach was used for exploratory purposes, and the results should be interpreted cautiously. Bootstrapping was not performed in the logistic regression analysis because of the large sample size and the fact that the number of events per variable exceeded 20 in the binary outcome. Instead, ROC curve analysis was used to assess how well the models distinguished between higher and lower frailty probability. This analysis provided area under the curve (AUC) estimates for the selected variables and allowed comparison of their discriminatory performance.

Multicollinearity was assessed to determine whether some independent variables carried overlapping information that could make coefficient estimates unstable. Collinearity in both the multiple and logistic regression models was evaluated using variance inflation factors (VIFs). Values of 5 or higher were considered indicative of problematic collinearity. In this study, no relevant multicollinearity was detected, as all VIF values were below 2.0. A summary of this analysis is provided in Multimedia Appendix 2.

Figure 1 shows a flowchart summarizing the analysis steps. As proof of concept, we conducted this preliminary analysis to assess the feasibility of the proposed approach. This analysis was not intended to provide definitive inference, but rather to demonstrate that the proposed method can be implemented and yield meaningful results under realistic conditions.

**Figure 1.**
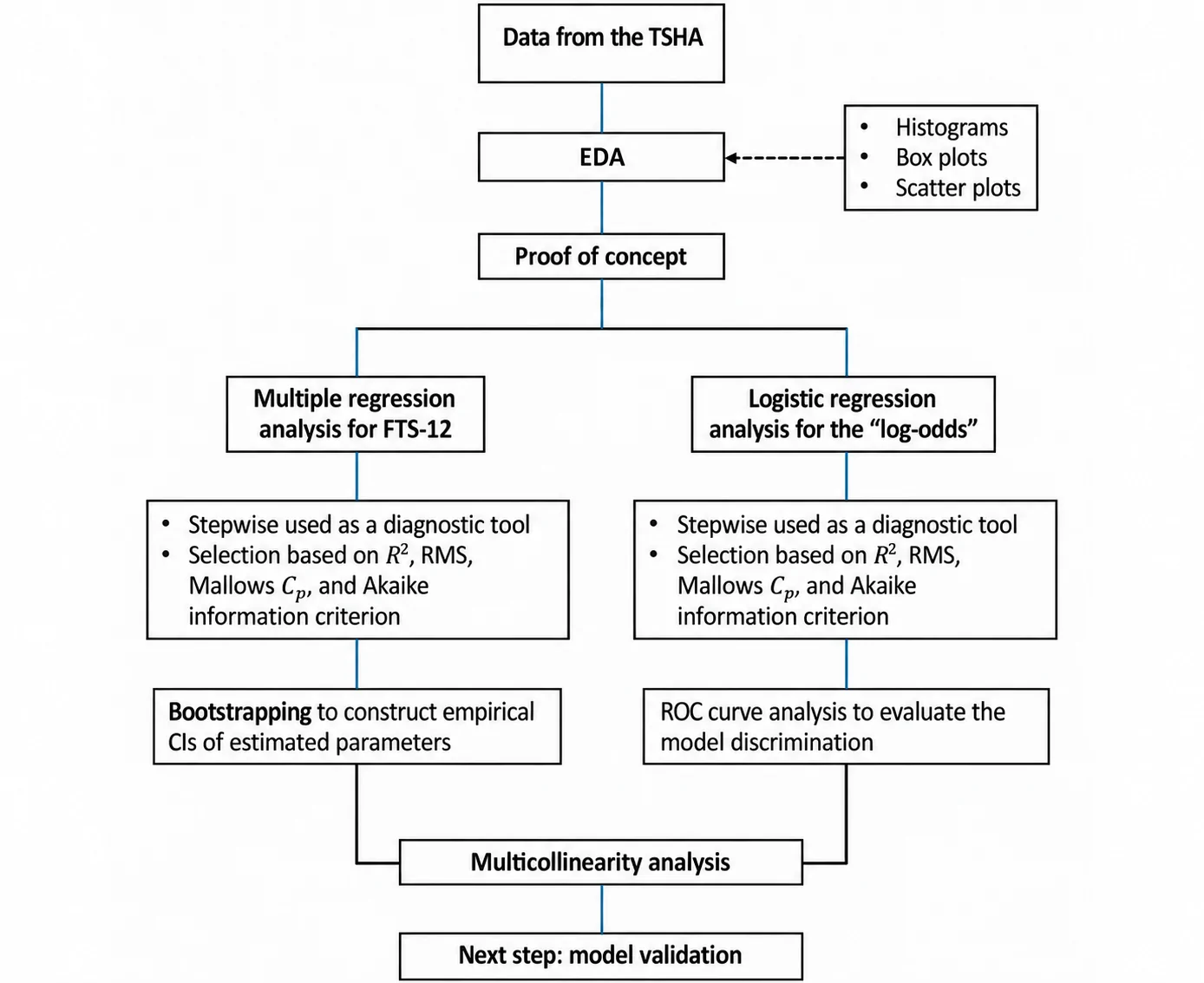
Flowchart summarizing the methodological workflow of the proof-of-concept study using data from the TSHA. EDA included histogram, box plot, and scatter plot visualizations. The FTS-12 score was analyzed using multiple linear regression, while the binary log-odds outcome was analyzed using logistic regression. A stepwise variable selection method was applied as an exploratory diagnostic tool, together with added model selection criteria. Bootstrapping procedures were incorporated into the multiple regression analysis, and ROC curve analysis was applied within the logistic regression framework. Multicollinearity assessment was conducted for both regression approaches. External model validation is pending and will be addressed in future work to further improve the variable selection process. EDA: exploratory data analysis; FTS-12: Frailty Trait Scale 12; RMS: residual mean square; ROC: receiver operating characteristic; TSHA: Toledo Study for Healthy Aging.

## Results

The results presented in this section correspond to an exploratory, proof-of-concept analysis and should be read accordingly: the estimates and discriminatory measures reported are intended to characterize the frailty-related signal contained in routine laboratory data, not to define validated clinical prediction tools. Their interpretation is therefore conditional on external validation in independent cohorts.

### Age and FTS-12 Values

The mean age of participants included in the selection process was 75.04 (SD 5.84; range 65‐95) years for female participants and 74.95 (SD 5.63; range 65‐99) years for male participants. The mean FTS-12 scores observed in the study were 46.68 (SD 15.99; range 6‐92.11) for female participants and 39.62 (SD 15.43; range 0‐84) for male participants. The original database comprised 847 (57%) female participants and 637 (43%) male participants.

### Models Based on FTS-12 Values: Selected Risk Factors and Functions

Tables S1 and S2 in Multimedia Appendix 3 summarize the variables retained in the regression models for female participants and male participants, respectively. In both sexes, the selected models combined individual laboratory markers with composite functions derived from clinically relevant variables. Table 1 presents the diagnostic statistics for the models stratified by sex. The *F*-statistics for both models were highly significant (*P*<.001), and the root mean square error ranged from 12 to 12.45. These findings highlight the relevance of both individual risk factors and their combined effects in modeling FTS-12 scores. Overall model performance was moderate, with *R*^2^ values of approximately 0.39 in female participants and 0.38 in male participants. The parameter estimates and associated statistics for the selected multiple regression models in female and male participants are presented in Tables 2 and 3, respectively. The definitions and mathematical expressions of the composite risk factors used across the regression models are provided in Table 4.

Figures 2 and 3 further illustrate the modeling quality of the models. A moderately strong Pearson correlation was observed between the observed and modeled FTS-12 scores for both sexes, with correlations of 0.621 for female participants and 0.615 for male participants. The predicted FTS-12 scores were reasonably aligned with the observed scores in both sexes, supporting the feasibility of this approach while also indicating that substantial prediction uncertainty remains. The scatter plots visually represent this positive relationship, with a colored ellipsoid enclosing 90% of the data points. Higher correlations indicate better agreement between observed and predicted FTS-12 values.

**Table 1.** Descriptive characteristics and diagnostic analysis of selected multiple regression models for female and male participants^a^.

Statistics	Case study	
	Female	Male
*F* value	46.81 (10, 744)	37.22 (9, 550)
*P* value	<.001	<.001
Root MSE^b^	12.45	12.00
*R* ^2^	0.39	0.38
Percentile 97.5% for *R*^2^	0.436	0.493
Percentile 2.5% for *R*^2^	0.298	0.312
Adjusted *R*^2^	0.38	0.37
Total of observations read	847	637
Total of observations used	755	560
Simple Pearson correlation between observed and expected values of FTS-12^c^	0.621	0.615

a Percentiles 97.5% and 2.5% for *R*2 were obtained by using the Bootstrapping technique. See the Modeling Regression Equations to Predict the FTS-12 Score section for further details.

b MSE: mean square error obtained from the ANOVA table of regression analyses.

c FTS-12: Frailty Trait Scale 12.

**Table 2.** Parameter estimates for the selected multiple regression model: female participants^a^.

Variable	Estimates					Correlations between FTS-12^b^ versus risk factors in the model		
	*df*	Estimate	SE	*t* test (*df*=744)	*P* value	Pearson correlation	*P* value	Values, n
Intercept	1	−337.16	32.57	−10.35	<.001	—^c^	—	—
CRF1^d^	1	25.40	2.16	11.74	<.001	0.578	<.001	825
CRF2	1	19.67	3.96	4.97	<.001	0.465	<.001	839
GGT^e^ (U/L)	1	0.1	0.037	3.18	.002	0.120	<.001	829
Urea (mg/dL)	1	0.15	0.048	3.17	.002	0.178	<.001	829
Granulocytes (%)	1	0.16	0.053	3.05	.002	0.201	<.001	838
Creatinine (mg/dL)	1	−9.25	3.16	−2.93	.004	0.132	<.001	840
Bilirubin total (mg/dL)	1	−5.40	1.96	−2.76	.006	−0.051	.14	839
Uric acid (mg/dL)	1	1.11	0.42	2.62	.009	0.187	<.001	840
Chlorine (mEq/L)	1	−0.30	0.16	−1.92	.05	−0.126	<.001	842
Triglycerides (mg/dL)	1	0.02	0.01	1.80	.07	0.068	.05	823
Age (years)	—	—	—	—	—	0.515	<.001	847
RDW^f^ (%)	—	—	—	—	—	0.240	<.001	828
Albumin (g/dL)	—	—	—	—	—	−0.389	<.001	843

a CRF1 and CRF2 are composite risk factors defined in Table 4. Simple Pearson correlations between each risk factor in the selected reduced model and the FTS-12 score are also shown.

b FTS-12: Frailty Trait Scale 12.

c Not applicable.

d CRF: composite risk factor.

e GGT: gamma-glutamyl transferase.

f RDW: red cell distribution width.

**Table 3.** Parameter estimates for the selected multiple regression model: male participants^a^.

Variable	Estimates					Correlations between FTS-12^b^ versus risk factors in the model		
	*df*	Estimate	SE	*t* test (*df*=550)	*P* value	Pearson correlation	*P* value	Values, n
Intercept	1	122.10	23.62	5.17	<.001	—^c^	—	—
CRF3^d^	1	−37.37	4.14	−9.02	<.001	−0.552	<.001	631
CRF2	1	15.06	5.10	2.95	.003	0.469	<.001	625
Chlorine (mEq/L)	1	−0.54	0.17	−3.19	.002	−0.111	.006	629
GGT^e^ (U/L)	1	−0.087	0.032	−2.68	.008	−0.128	.001	620
Triglycerides (mg/dL)	1	0.033	0.012	2.63	.009	0.009	.82	617
Uric acid (mg/dL)	1	0.94	0.39	2.41	.02	0.080	.046	625
Phosphorus (mg/dL)	1	2.48	1.04	2.37	.02	0.048	.23	626
Eosinophil (%)	1	−0.56	0.29	−1.90	.05	−0.040	.32	623
Leukocytes (×10^3^/µL)	1	0.60	0.33	1.86	.06	0.090	.02	626
Age (years)	—	—	—	—	—	0.476	<.001	637
Albumin (g/dL)	—	—	—	—	—	−0.427	<.001	631
Alkaline phosphatase (U/L)	—	—	—	—	—	0.152	.001	628

a CRF2 and CRF3 are composite risk factors defined in Table 4. Simple Pearson correlations between each risk factor in the selected reduced model and the FTS-12 score are also shown.

b FTS-12: Frailty Trait Scale 12.

c Not available.

d CRF: composite risk factor.

e GGT: gamma-glutamyl transferase.

**Table 4.** Definitions of composite risk factors (CRFs) used in the regression models^a^.

Label	Descriptor	Formula	Selected in model
CRF1	RDW^b^-age-albumin composite	ln(RDW×age^3^/albumin)	Female participants, multiple regression
CRF2	Alkaline phosphatase-age-albumin composite	ln(AlkPhos^c^×age/Albumin)	Female and male participants, multiple regression
CRF3	Albumin-age composite	ln(albumin)×00/age	Male participants, multiple regression; female participants, logistic regression
CRF4	Hemoglobin-age composite	Hemoglobin×10^5^/age^3^	Male participants, logistic regression

a Logarithmic transformations were applied to better capture nonlinear relationships between predictors and the Frailty Trait Scale 12 score, consistent with standard practice in regression modeling [21]. CRF identifiers are used throughout the paper; full mathematical definitions appear only in this table.

b RDW: red cell distribution width.

c AlkPhos: alkaline phosphatase.

**Figure 2.**
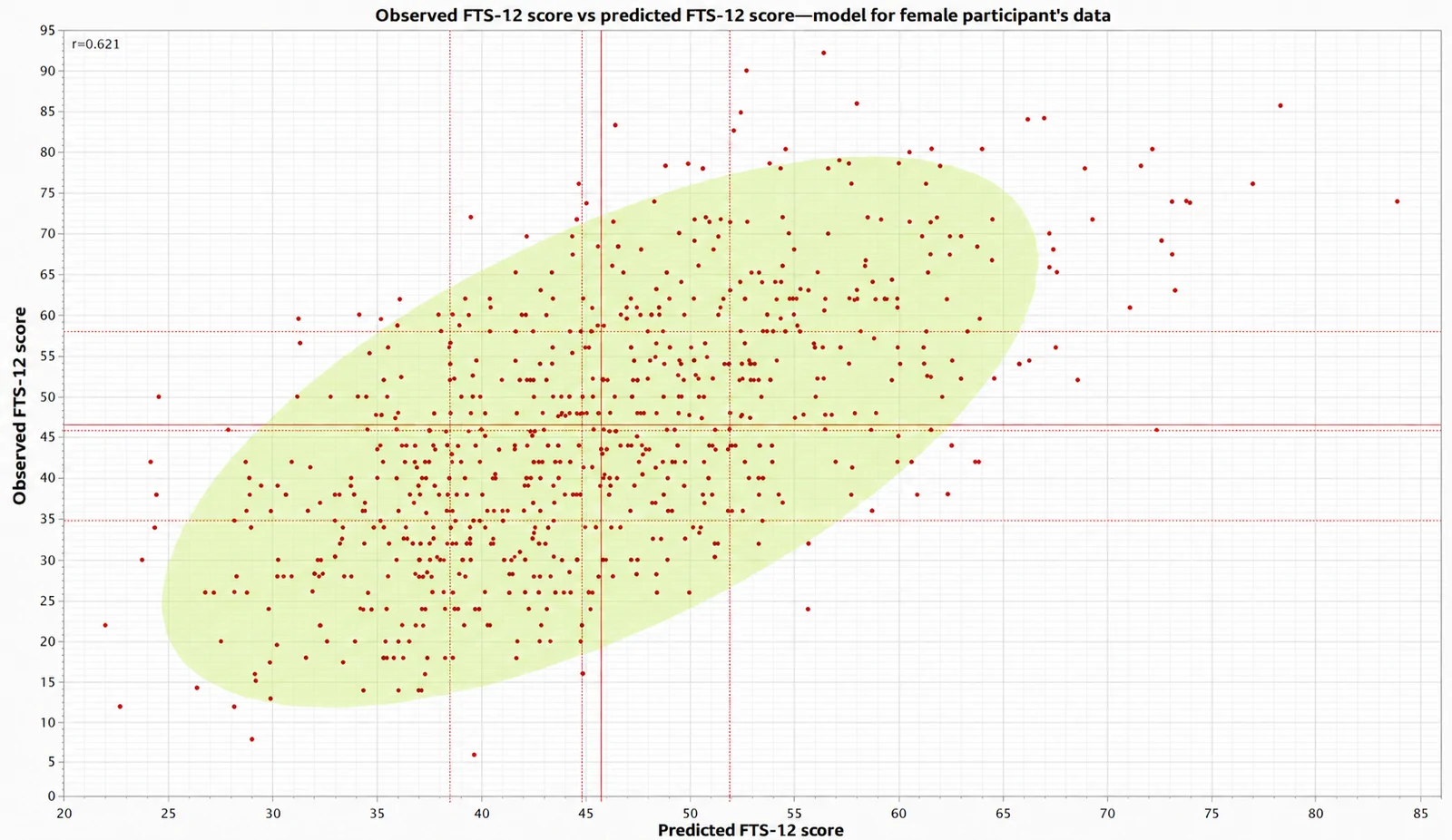
Observed versus modeled FTS-12 score using the selected multiple regression model in female participants (n=848). The horizontal axis shows the FTS-12 score predicted by the sex-specific multiple regression model (which retained age, albumin, gamma-glutamyl transferase, and selected composite risk factors as predictors; Table 2), and the vertical axis shows the FTS-12 score observed for each participant. The simple Pearson correlation was 0.621 (*P*<.001). Continuous vertical and horizontal red lines show the mean values for the modeled and observed FTS-12. Dashed vertical and horizontal red lines are the first (lower), second (median), and third (upper) quartiles. The shaded ellipse is the region encompassing 90% of the plotted data. A higher correlation, and closer alignment of points along the diagonal, indicate better agreement between observed and modeled FTS-12 values. The moderate dispersion observed is consistent with the multidimensional nature of frailty, only part of which is captured by routine laboratory data. FTS-12: Frailty Trait Scale 12.

**Figure 3.**
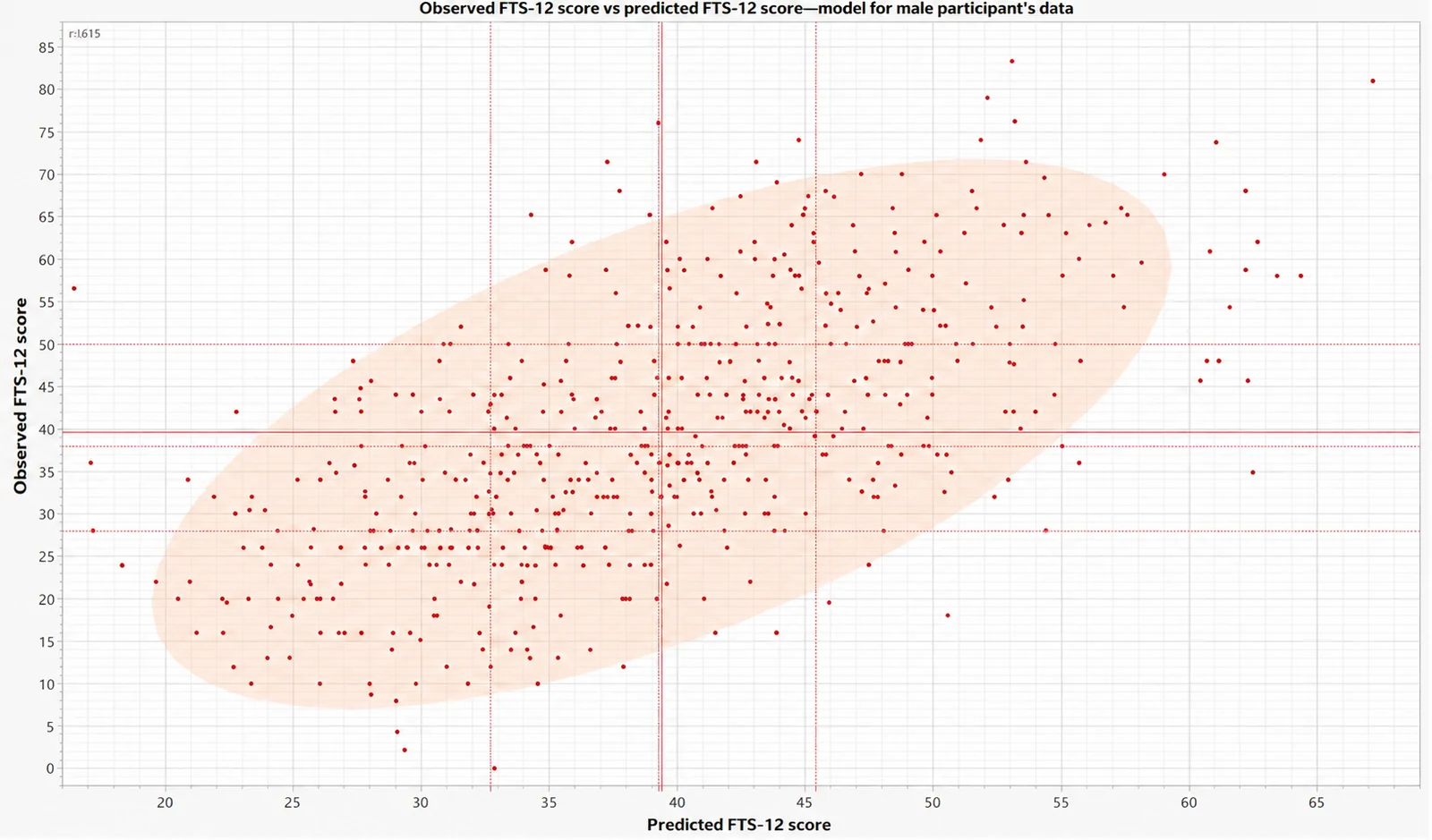
Observed versus modeled FTS-12 scores using the selected multiple regression model in male participants (n=637). The horizontal axis shows the FTS-12 score predicted by the sex-specific multiple regression model (which retained age, albumin, gamma-glutamyl transferase, and a composite risk factor as predictors; Table 3), and the vertical axis shows the FTS-12 score observed for each participant. The simple Pearson correlation was 0.615 (*P*<.001). Continuous vertical and horizontal red lines show the mean values for the modeled and observed FTS-12. Dashed vertical and horizontal red lines are the first (lower), second (median), and third (upper) quartiles. The shaded ellipse is the region encompassing 90% of the plotted data. A higher correlation, and closer alignment of points along the diagonal, indicate better agreement between observed and modeled FTS-12 values. The moderate dispersion observed is consistent with the multidimensional nature of frailty, only part of which is captured by routine laboratory data. FTS-12: Frailty Trait Scale 12.

### Regression Analysis of Statistical Models

Tables 2 and 3 present the regression parameter estimates and related statistics for the 2 selected models. The composite risk functions were more strongly related to the FTS-12 score than most single laboratory variables. This suggests that frailty is better captured by combinations of biologically related markers than by isolated biomarkers alone. In female participants, the strongest association was observed for the function combining red cell distribution width (RDW), age, and albumin, whereas in male participants, the strongest association involved albumin- and age-related composite terms. These findings support the idea that frailty risk is influenced by interacting physiological processes rather than by a single laboratory abnormality. To enhance visualization, colored ellipsoids enclosing 90% of the data points are included in both figures. These ellipsoids highlight the trends that informed the selection of these functions for the models. Notably, the relationship is not strong enough to approximate a straight line. If they were, each value of the function composite risk factor (CRF) 1 (Figure S1 in Multimedia Appendix 4) or CRF3 (Figure S2 in Multimedia Appendix 4) would correspond to only 1 or a few closely related FTS-12 values. Instead, the ellipsoids suggest a broader range of FTS-12 scores for a specific value of each function. Nonetheless, a positive trend is evident for CRF1 (Figure S1 in Multimedia Appendix 4), while a negative trend is observed for CRF3 (Figure S2 in Multimedia Appendix 4). Comparatively, correlations with single risk factors were moderate. For female participants, the highest correlation was 0.187 (*P*<.001) for uric acid, with a positive correlation observed for gamma-glutamyl transferase (GGT; 0.120; *P*=.001). In contrast, GGT showed a negative correlation in male participants (−0.128; *P*=.001).

Correlations between FTS-12 and key variables such as age, RDW, albumin, and alkaline phosphatase were also examined. Age and albumin were of particular interest, as they were included in all CRF functions, regardless of sex. For age, the correlation was 0.515 (*P*<.001) in female participants and 0.476 (*P*<.001) in male participants. For albumin, the correlation was −0.389 (*P*<.0001) in female participants and −0.427 (*P*<.001) in male participants. Despite its moderately high correlation, albumin was not selected as an “individual variable.” Plausible reasons for this exclusion are discussed later in the text.

### Models Based on High Frailty Probability: Selected Risk Factors and Functions

Tables S3 and S4 in Multimedia Appendix 3 summarize the statistics for the predicted probabilities (hereafter referred to as “the probabilities”) and the independent variables, including individual risk factors and composite functions, incorporated into the selected logistic models for female participants and male participants, respectively. These tables also include information on “age” and several other risk factors. Although these variables were not selected as individual predictors, they are components of the CRF functions named through the selection process.

Table 5 provides descriptive statistics and diagnostic parameters for the selected logistic models. Among the participants, 200 of 735 (27%) in the female participants and 100 of 564 (18%) in the male participants had FTS-12 scores in the fourth quarter (above the third quartile, showing a high probability of frailty). The remaining participants in each group were distributed across the lower 3 quarters.

**Table 5.** Descriptive characteristics and diagnostic analysis of selected logistic regression models for female participants and male participants.

Statistics	Case study	
	Female participants	Male participants
Observations read, n	847	637
Observations used, n	735	564
Observations with an FTS-12^a^ value located in the fourth interval (larger than the third quartile), n	200	100
Observations with an FTS-12 value not located in the fourth interval (larger than the third quartile), n	535	464
Model fit statistics criterion		
AIC^b^	696.4	421.8
−2 Log *L*	682.4	403.8
Max-rescaled *R*^2^	0.312	0.323
Testing global null hypothesis: only intercept		
Likelihood ratio		
Chi-square (*df*)	178.0 (6)	123.3 (10)
*P* value	<.001	<.001

a FTS-12: Frailty Trait Scale 12.

b AIC: Akaike information criterion.

Tests of the global null hypothesis were highly significant (*P*<.001), showing that at least 1 independent variable—whether a single risk factor or a composite function of risk factors—was significantly associated with the probability of FTS-12 falling into the uppermost range.

Tables 6 and 7 summarize the estimated parameters and associated statistics for the selected models applied to female and male participants, respectively. The model for female participants includes 5 single variables and 1 composite function of risk factors. In contrast, the model for male participants comprises 7 single variables and 1 composite function of risk factors.

**Table 6.** Parameter estimates for the selected logistic model: female participants^a^.

Variable	Estimates					Correlations between probability versus risk factors in the model			Odds ratio estimates
	*df*	Estimate	SE	Wald chi-square (*df=*1)	*P* value	Pearson correlation	*P* value	Values, n	
Intercept	1	7.83	1.43	30.0	<.001	—^b^	—	—	—
CRF3^c^	1	−6.21	0.63	96.9	<.001	−0.871	<.001	735	0.00
Granulocytes (%)	1	0.035	0.011	10.2	.001	0.349	<.001	735	1.04
Alkaline phosphatase (U/L)	1	0.011	0.004	5.8	.02	0.333	<.001	735	1.01
Urea (mg/dL)	1	0.012	0.0082	2.1	.14	0.248	<.001	735	1.01
LDL^d^ (mg/dL)	1	−0.0057	0.0031	3.4	.07	−0.196	<.001	735	0.99
GGT^e^ (U/L)	1	0.021	0.0070	8.9	.003	0.185	<.001	735	1.02
Age	—	—	—	—	—	0.809	<.001	735	—
FTS-12^f^	—	—	—	—	—	0.580	<.001	735	—

a Simple Pearson correlations between each risk factor included in the selected model and the estimated probability of the FTS-12 values greater than the third quartile (fourth quarter) are also presented. CRF3 is a composite risk factor defined in Table 4. Pearson correlations between each risk factor and the modeled probability of falling above the FTS-12 third quartile are also presented, together with odds ratio estimates.

b Not available.

c CRF: composite risk factor (Table 4).

d LDL: low-density lipoprotein.

e GGT: gamma-glutamyl transferase.

f FTS-12: Frailty Trait Scale 12.

**Table 7.** Parameter estimates for the selected logistic model: male participants^a^.

Variable	Estimates					Correlations between probability versus risk factors in the model			Odds ratio estimates
	*df*	Estimate	SE	Wald chi-square (*df=*1)	*P* value	Pearson correlation	*P* value	Values, n	
Intercept	1	11.16	5.27	4.5	.03	—^b^	—	—	—
CRF4^c^	1	−1.28	0.21	36.8	<.001	−0.641	<.001	564	0.19
Albumin	1	−2.70	0.53	26.2	<.001	−0.623	<.001	564	0.07
Alkaline phosphatase (U/L)	1	0.013	0.0054	6.0	.01	0.299	<.001	564	1.00
CHCM^d^ (g/L)	1	−0.28	0.16	3.0	.08	−0.192	<.001	564	0.76
Hemoglobin (g/dL)	1	0.46	0.11	16.4	<.001	−0.190	<.001	564	1.58
Triglycerides (mg/dL)	1	0.012	0.0029	16.2	<.001	0.182	<.001	564	1.01
Phosphorus (mg/dL)	1	0.89	0.28	10.3	.001	0.134	<.001	564	2.43
Lactate dehydrogenase (U/L)	1	0.0017	0.0021	0.6	.42	0.057	.001	564	1.00
Age	—	—	—	—	—	0.707	<.001	564	—
FTS-12^e^	—	—	—	—	—	0.538	<.001	564	—

a Simple Pearson correlations between each risk factor included in the selected model and the estimated probability of the FTS-12 values greater than the third quartile of the distribution are also presented. CRF4 is a composite risk factor defined in Table 4. Pearson correlations between each risk factor and the modeled probability of falling above the FTS-12 third quartile are also presented, together with odds ratio estimates.

b Not applicable.

c CRF: composite risk factor (Table 4).

d CHCM: mean corpuscular hemoglobin concentration.

e FTS-12: Frailty Trait Scale 12.

Considering the Wald chi-square statistics, the presence of CRF3, granulocytes, and GGT is highly significant and essential in the model for female participants. For male participants, CRF4, albumin, hemoglobin, triglycerides, and phosphorus are identified as highly significant and critical associated factors. Tables 6 and 7 also present Pearson correlations between each selected independent single variable or composite function of risk factors and the modeled probability. Additionally, these tables include correlations between the modeled probability and both age and FTS-12 scores within the working datasets. In absolute terms, correlations involving composite functions of risk factors exceeded 0.64 (*P*<.001). Notably, the function CRF3 in female participants showed particularly strong correlations. Among the single risk factors, albumin showed a highly negative correlation in the selected model for male participants.

Figures S3 and S4 in Multimedia Appendix 4 illustrate the functional relationship between the modeled probability and the composite functions of risk factors with the highest correlations. However, these associations were nonlinear. For the logistic model applied to female participants, the relationship between the observed values of the function and the predicted probability followed an inverted logistic S-curve (Figure S3 in Multimedia Appendix 4), with a simple correlation of −0.871 (*P*<.001). When the value of the function exceeded 2.20, the probability fell below .20 (odds of 1 to 4). Conversely, when values of the function were less than 1.55, the probability exceeded .70 (odds of 7 to 3).

Similarly, for the logistic model in male participants, the relationship between function CRF4 and the modeled probability also followed an inverted logistic S-curve (Figure S4 in Multimedia Appendix 4), with a simple correlation of −0.641 (*P*<.001). Here, values of the function above 4.75 corresponded to probabilities lower than .20 (odds of 1 to 4), whereas values below 1.50 were associated with probabilities exceeding .70 (odds of 7 to 3). We also estimated the simple correlations between the modeled probability and both patient age and observed FTS-12 values in the original datasets. Age was consistently included in all composite functions of risk factors selected by the selection process. All correlations were highly significant (*P*<.001), irrespective of sex. Notably, correlations with age were stronger than those with FTS-12 (Tables 6 and 7).

Multimedia Appendix 4 also illustrates the functional relationship between the modeled probability and age in the selected models. For female participants, the scatter plot in Figure S5 in Multimedia Appendix 4 demonstrates that the distribution of values aligns closely with a logistic S-curve. Similarly, for male participants, Figure S6 in Multimedia Appendix 4 exhibits a positive trend, also resembling a logistic S-curve.

Positive correlations between the observed FTS-12 values and the modeled probabilities are clearer in the scatter plots presented in Figures 4 and 5 for male and female participants, respectively. While the dispersion of points in both plots shows a moderate correlation, a clear trend appears, highlighting the prediction capability of the selected models. This consistency in predictive performance is clear across both sexes.

**Figure 4.**
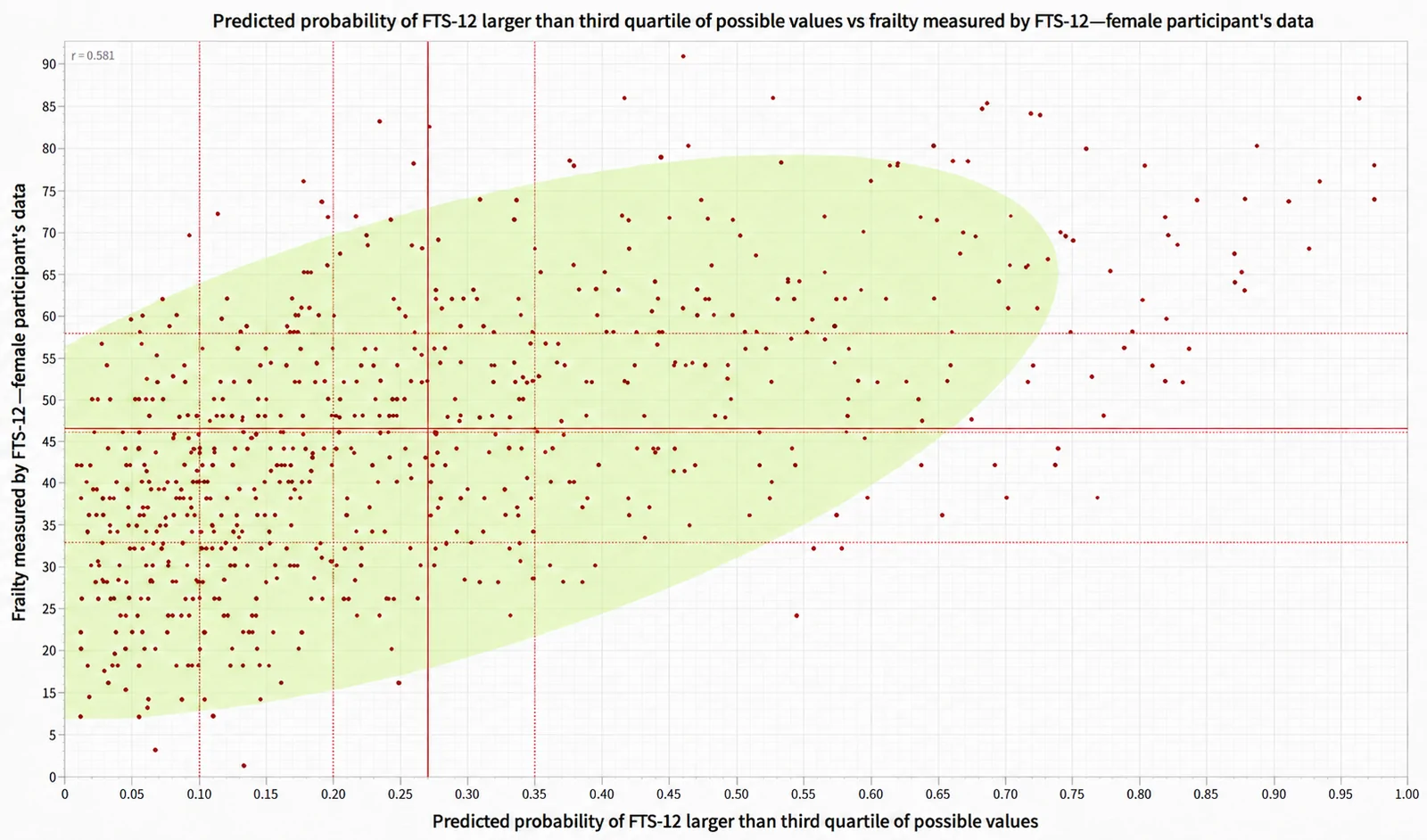
Scatter plot of observed FTS-12 values versus the modeled probability of the frailty scores larger than the third quartile of the distribution of possible FTS-12 values, based on the selected logistic model in female participants (n=848). The horizontal axis shows the modeled probability returned by the sex-specific logistic regression model (which retained albumin- and age-based composite risk factors and selected single biomarkers as predictors; Table 6), and the vertical axis shows the observed FTS-12 score for each participant. The probability quantifies the model-estimated likelihood that an individual falls within the highest-burden frailty subgroup of the cohort (ie, above the FTS-12 third quartile), used here as an internal analytical cutoff rather than as a clinical threshold. The Pearson correlation coefficient is 0.581 (*P*<.001). Continuous red lines are the mean values of the observed FTS-12 values and the modeled probability, while dashed red lines mark the first (lower), second (median), and third (upper) quartiles. The shaded ellipse is the region encompassing 90% of the plotted data. FTS-12: Frailty Trait Scale 12.

**Figure 5.**
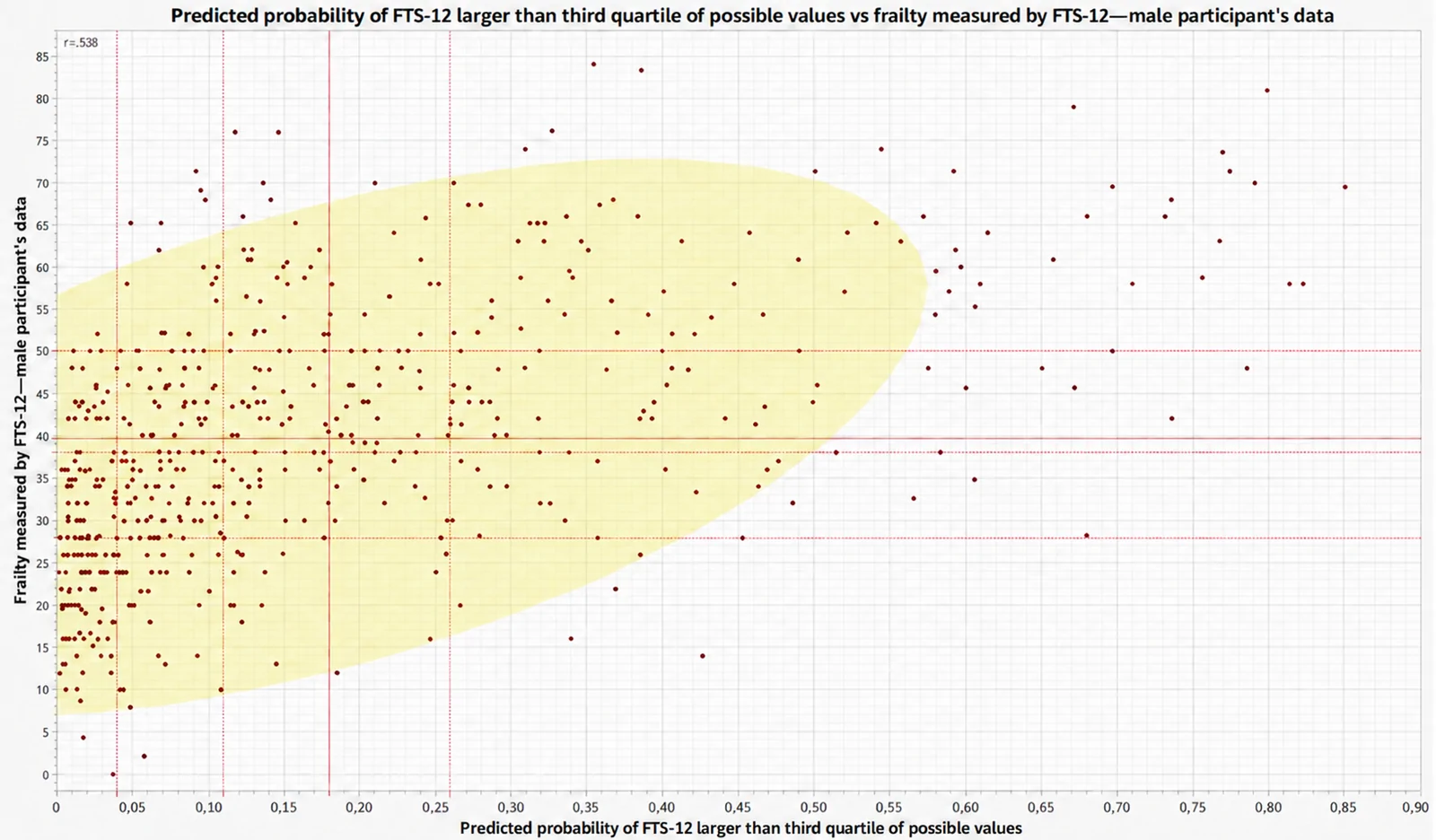
Scatter plot of observed FTS-12 values versus the modeled probability of the frailty scores larger than the third quartile of the distribution of possible FTS-12 values, based on the selected logistic model in male participants (n=637). The horizontal axis shows the modeled probability returned by the sex-specific logistic regression model (which retained hemoglobin- and age-based composite risk factors and selected single biomarkers as predictors; Table 7), and the vertical axis shows the observed FTS-12 score for each participant. The probability quantifies the model-estimated likelihood that an individual falls within the highest-burden frailty subgroup of the cohort (ie, above the FTS-12 third quartile), used here as an internal analytical cutoff rather than as a clinical threshold. The Pearson correlation coefficient is 0.538 (*P*<.001). Continuous red lines are the mean values of the observed FTS-12 values and the modeled probability, while dashed red lines mark the first (lower), second (median), and third (upper) quartiles. The shaded ellipse is the region encompassing 90% of the plotted data. FTS-12: Frailty Trait Scale 12.

### Discriminatory Performance of the Logistic Models (ROC Analysis)

Using the AIC and −2Log *L* (minus 2 log-likelihood) to assess goodness-of-fit, the model for male participants proved a better fit compared to the model for female participants. Additionally, chi-square statistics derived from the likelihood ratio test provided strong evidence that the independent variables significantly influenced both selected models. The intercept alone was insufficient to explain the observed log-odds—the logarithm of the ratio between the probability of a FTS-12 value being larger than the third quartile of the distribution, and the probability of it not doing so. In addition to goodness-of-fit metrics, ROC curve analysis was used to evaluate the discriminatory performance of the selected logistic models. Tables S5 and S6 in Multimedia Appendix 3 provide statistics from the ROC analysis of the selected logistic models for both sexes. The ROC curves align closely with the goodness-of-fit statistics. For female participants, the AUC was approximately 0.799. Table S5 in Multimedia Appendix 3 and Figure 6 show that the function CRF3 was the most influential risk factor, accounting for 76% of the total AUC and closely approximating the model’s overall AUC. For male participants, the AUC was approximately 0.824. Table S6 in Multimedia Appendix 3 and Figure 7 reveal that CRF4 and albumin were the most influential risk factors, contributing 73% and 71% of the total AUC, respectively.

**Figure 6.**
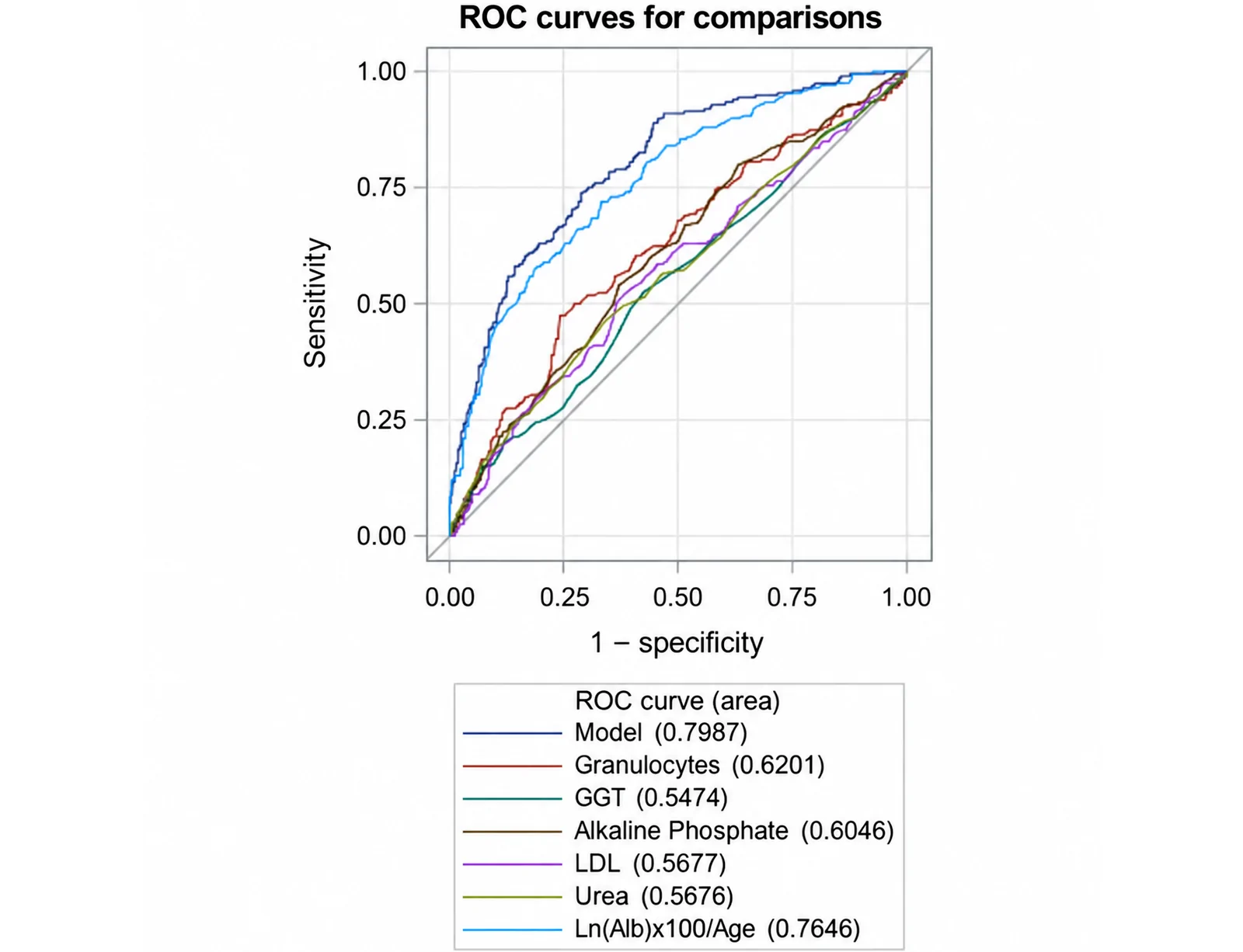
ROC curves for the selected logistic regression model and partial models in female participants (n=848). The ROC curve for the selected logistic regression model is shown alongside the ROC curves of partial models that include individual or composite risk factors for comparison. The curves illustrate the discriminatory power for the log-odds (and related probability) of an FTS-12 score in the uppermost range (above the third quartile) of the cohort. The horizontal axis shows 1–specificity (false positive rate), and the vertical axis shows sensitivity (true positive rate); the diagonal represents a noninformative classifier. The AUC for the selected model was 0.799, indicating moderate-to-good discrimination, and was substantially higher than the AUC of any model built on a single biomarker. The largest contribution among the partial models came from CRF3, accounting for approximately 76% of the total AUC. Key statistics, including the AUC for each model, highlight the improvement in predictive performance when combining risk factors in the selected model. CRF3 (the albumin-age composite) is defined in Table 4. AUC: area under the curve; CRF: composite risk factor; FTS-12: Frailty Trait Scale 12; GGT: gamma-glutamyl transferase; LDL: low-density lipoprotein; ROC: receiver operating characteristic.

**Figure 7.**
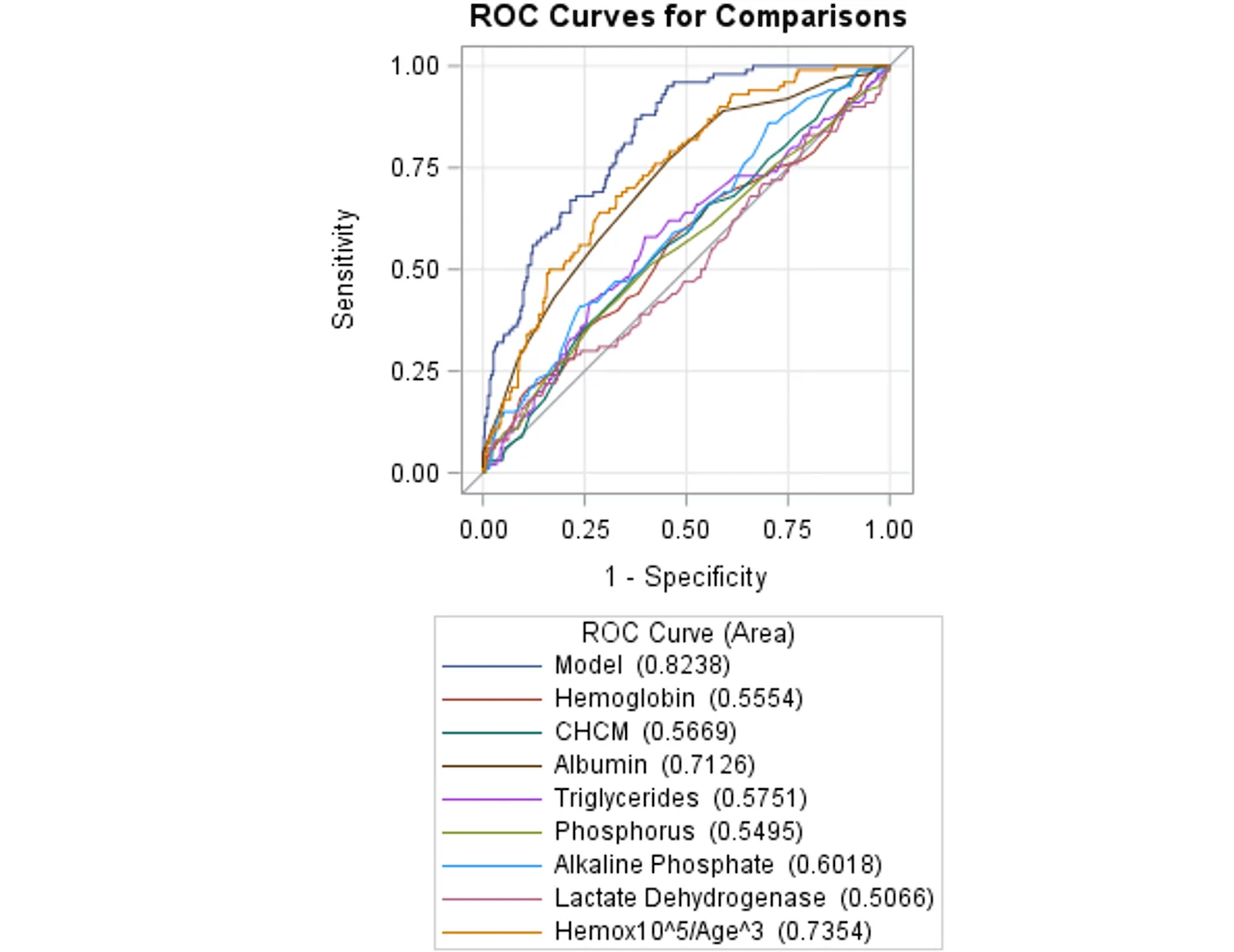
ROC curves for the selected logistic regression model and partial models in male participants (n=637). The ROC curve for the selected logistic regression model is shown alongside the ROC curves of partial models that include individual or composite risk factors for comparison. The curves illustrate the discriminatory power for the log-odds (and related probability) of an FTS-12 score in the uppermost range (above the third quartile) of the cohort. The horizontal axis shows 1–specificity (false positive rate), and the vertical axis shows sensitivity (true positive rate); the diagonal represents a noninformative classifier. The AUC for the selected model was 0.824, indicating moderate-to-good discrimination, and was substantially higher than the AUC of any model built on a single biomarker. The largest individual contributions to the model AUC came from CRF4 (approximately 73% of the total AUC) and albumin (approximately 71%). Key statistics, including the AUC for each model, highlight the improvement in predictive performance when combining risk factors in the selected model. CRF4 (the hemoglobin-age composite) is defined in Table 4. AUC: area under the curve; CHCM: mean corpuscular hemoglobin concentration; CRF: composite risk factor; FTS-12: Frailty Trait Scale 12; ROC: receiver operating characteristic.

## Discussion

### Principal Findings

This exploratory study aimed to identify and refine sex-specific predictive equations for the FTS-12 score using routinely available biochemical and hematological data, including an analytical strategy to estimate the likelihood of belonging to the highest frailty range of the cohort. In line with these aims, the study showed that sex-specific multiple regression models could estimate the FTS-12 score with moderate accuracy, and that sex-specific logistic models could identify predictors associated with being in the upper range of the FTS-12 distribution. Across both approaches, age, albumin, GGT, and several composite variables emerged as recurrent contributors, suggesting that frailty-related laboratory signals are better captured through combinations of markers than through most single biomarkers alone. Taken together, these findings support the feasibility of a low-cost, laboratory-based approach for exploratory frailty risk stratification, but the present models do not constitute validated clinical prediction tools and would require external validation in independent cohorts before any clinical application can be considered.

### Interpretation of Findings and Comparison With Previous Literature

Our findings suggest that routinely available biochemical and hematological data can provide a meaningful, although still imperfect, estimate of frailty burden as measured by the FTS-12 score, as well as of the likelihood of being in the highest frailty range of the cohort. Some uncertainty in prediction is expected, as the FTS-12 reflects a multidimensional construct integrating 7 dimensions, including energy balance, nutrition, and vascular system components [11], rather than a single biological process. In this context, combinations of laboratory variables performed better than most isolated biomarkers, consistent with the stronger associations observed for the selected composite risk functions in both sexes (Tables 2 and 3 and Figures S3 and S4 in Multimedia Appendix 4). The logarithmic transformations applied to variables such as albumin, age, and hemoglobin should be understood as a way to capture nonlinear clinical relationships and improve model fit, rather than as an end in themselves [21]. For example, combinations involving age and albumin may reflect the joint contribution of aging-related vulnerability and reduced nutritional or systemic reserve, in line with literature linking low albumin and hypoproteinemia to frailty, inflammation, and nutritional vulnerability in older adults [22,23]. Likewise, functions including RDW, hemoglobin, or albumin may capture overlapping signals related to erythrocyte heterogeneity, oxygen-carrying capacity, anemia-related vulnerability, and physiological resilience, consistent with evidence associating RDW and lower hemoglobin status with frailty in older populations [24,25]. In the logistic models, composite risk functions showed the strongest associations with higher frailty probability; and in male participants, albumin also emerged as an important contributor, supporting the interpretation of frailty as a multidimensional state of vulnerability shaped by interacting biological processes.

The models explained about 40% of the variability in FTS-12 scores, indicating moderate predictive performance. This level of explanation is clinically meaningful for an initial screening-oriented approach, while also showing that a substantial part of frailty remains influenced by factors not captured in laboratory-based models—a multidimensional syndrome reflecting biological, functional, cognitive, psychosocial, and environmental factors. The unexplained variance is therefore expected and reinforces that these models should be viewed as complementary tools rather than substitutes for comprehensive geriatric assessment, with future improvements depending on incorporating additional clinical, behavioral, and social determinants of frailty. Notably, albumin and age emerged as key associated factors consistently included across all composite functions, with logarithmic transformations capturing nonlinear relationships more effectively than models based solely on individual risk factors.

Recent literature includes only a few studies that model frailty risk using biochemical traits in older adults [21,23,26]. Several recent works have developed statistical models for frailty and frailty-associated outcomes such as mortality, most of them using logistic regression [27-30]. For example, Bencivenga et al [31] examined a FI composed of 38 multidimensional health deficits—including comorbidities, laboratory and diagnostic data, and symptoms and signs of disease—using procedures outlined by Searle et al [32]; the independent variables in their multiple regression model included sex (binary), age, BMI, and muscle thickness of the vastus intermedius and rectus femoris. Logistic regression is a widely used technique for modeling binary dependent variables in this setting [33-35]. Wang et al [36] provide a recent example using logistic regression and ROC curve analysis to compare 2 FIs in classifying binary mortality outcomes: the first based on routine physical assessments and laboratory tests, the second on self-reported data encompassing 30 health problems (“health deficits”) such as diseases, symptoms, disabilities, and psychological issues. In their study, FI values were used as independent variables to model the log-likelihood of dying. The clinical relevance of laboratory-based frailty stratification was further supported by Pasternak et al [37], who demonstrated that a laboratory-based FI was significantly associated with all-cause mortality in hospitalized older adults.

In contrast, our study used FTS-12 measurements to generate a new binary dependent variable, categorizing scores as exceeding the third quartile of the distribution or not. This allowed us to model the likelihood that an observed FTS-12 value would fall within the uppermost range, with logistic regression then applied to assess the log-likelihood of these outcomes. Liu et al [38](2021) also used logistic regression, but with a different approach: a binary outcome representing the presence or absence of frailty in older patients with nutritional risks, with frailty status determined from previous research. However, Liu et al [38] focused on exogenous (environmental) predictors such as age, sex, ethnicity, educational level, BMI, walking ability, activities of daily living, and cognitive functioning, whereas our study prioritized “endogenous” biochemical variables to identify both the “most reasonable” model and the “most influential” risk factors.

Álvarez-Sánchez et al [39] used logistic regression to evaluate the association between 2 inflammatory biomarkers—high-sensitivity C-reactive protein and homocysteine—and prefrailty in Spanish adults aged 65 years and older [3], underscoring the role of inflammation in age-related physical decline. Although neither biomarker was selected in our models, our findings on GGT and albumin are consistent with emerging geriatric evidence. In a Korean population-based cohort, Kang et al [26] reported that elevated GGT levels were 26% higher in frail male participants than in nonfrail male participants, with higher GGT quartiles showing a 2.08-fold increased odds of frailty. Elevated GGT has also been associated with poorer muscle function—including reduced jump power and diminished handgrip strength—independent of body-composition factors [40], and with increased risk of hip fracture in postmenopausal female participants, irrespective of alcohol consumption or chronic liver disease [41]. Because both diminished handgrip strength and elevated risk of hip fracture are strongly associated with frailty in older adults, these observations support the potential relevance of GGT in frailty risk estimation [3,11]. A recent systematic review further emphasized that hypoalbuminemia is associated with a significant risk of frailty and adverse health consequences in hospital and community contexts [24]. Together, these findings strengthen the biological plausibility of our results, suggesting that GGT and albumin reflect convergent pathways of oxidative stress, malnutrition, and chronic inflammation that are central to geriatric syndromes [42].

From a model-performance perspective, the diagnostic criteria used to compare candidate logistic models suggested acceptable exploratory fit [20,33,43]. More importantly, from a clinical standpoint, the models showed that higher frailty probability was better captured by combinations of laboratory variables than by most isolated biomarkers alone. This supports the idea that frailty-related vulnerability reflects interacting biological domains rather than a single abnormal test result and helps explain why composite terms contribute more consistently than most individual variables. Notably, the strongest correlations were seen between CRF functions and both FTS-12 values and the associated probabilities, regardless of sex. However, substantial dispersion in observed FTS-12 values persisted for each value of the composite risk functions, highlighting the complex interplay of factors across the 7 dimensions underlying frailty assessment.

Placed in the context of existing approaches for laboratory-based frailty screening, the discriminatory performance reported in the Results section falls within the moderate-to-good range typically described for tools that rely exclusively on routine biochemical and hematological variables [11,21]. AUC values close to 0.80 for both sexes are consistent with what may reasonably be expected when functional, cognitive, and self-reported components are not incorporated, and they reinforce the view that the present models should be regarded as a complementary first layer of risk stratification rather than as a stand-alone diagnostic instrument. Beyond their overall discriminatory performance, the biological composition of the dominant composite functions is also informative: the prominence of the albumin-age composite in female participants is consistent with the well-established association of hypoalbuminemia and anemia with adverse outcomes in older adults [24,25], while the relevance of the hemoglobin-age composite in male participants is compatible, with erythropoietic decline acting as a sex-sensitive marker of physiological reserve. The recurrent involvement of GGT and alkaline phosphatase further suggests that subclinical hepatobiliary and oxidative pathways contribute to the laboratory signature of frailty [26], in line with the broader role of inflammatory, nutritional, and oxidative mechanisms previously implicated in the syndrome [3,39,42].

### Limitations

Variable selection was conducted using stepwise multiple regression guided by the AIC and Mallows *C*_p_ statistic. Although stepwise procedures have recognized limitations, this approach was used for exploratory purposes within a proof-of-concept framework, and the results should therefore be interpreted with appropriate caution.

This study has several limitations that should temper the interpretation of its findings. First, its cross-sectional design prevents causal inference and may be susceptible to reverse causation; for example, elevated GGT could reflect unmeasured liver disease or alcohol intake rather than etiological pathways to frailty [26,44]. Second, all biochemical measurements were obtained at a single baseline visit, so temporal fluctuations and dynamic changes in frailty status were not evaluated. Third, the cohort was drawn from a single European population (Toledo, Spain), which may limit external validity in ethnically diverse or resource-limited settings. Finally, model development and internal validation were performed using the same dataset, which may increase the risk of overfitting despite the application of bootstrapping procedures. Future studies should therefore prioritize external or independent validation to assess model performance on unseen data and the generalizability of the proposed models.

Furthermore, rather than serving as a general clinical cutoff for the FTS-12 scale, the binary logistic analysis based on the upper quartile should be understood as an internal analytical technique to identify the subgroup with the highest frailty burden within this cohort. We recognize that dichotomization may reduce information and affect apparent model discrimination. The current proof-of-concept study did not include formal calibration analyses (calibration plots, calibration slope/intercept, and Brier score), threshold-specific sensitivity and specificity, decision-curve analysis, or net-benefit evaluation, although discrimination was investigated using ROC curve analysis and multicollinearity was confirmed to be nonproblematic using VIFs.

As noted in the Methods section, calibration in the probabilistic sense did not apply to the multiple regression model, for which performance was instead evaluated through goodness-of-fit measures and residual diagnostics. For the logistic models, formal calibration was not assessed. The present models should therefore be interpreted as exploratory, discrimination-based tools to determine whether routinely available laboratory data contain meaningful frailty-related signals, rather than as deployment-ready clinical prediction instruments. Further development toward clinical implementation will require calibration-focused evaluation, external validation, and implementation-oriented testing. Although bootstrap procedures were used to estimate CIs for selected model parameters, this does not constitute a full internal validation; optimism-corrected evaluation of overall model performance was beyond the scope of this secondary exploratory analysis.

More stringent development and validation techniques—penalized regression (eg, least absolute shrinkage and selection operator), repeated cross-validation, formal calibration evaluation, clinical utility analyses, and benchmarking against parsimonious and machine-learning alternatives—should be incorporated into model refinement in later phases. These procedures were outside the scope of the current work because the study was not preregistered and was intended as a secondary exploratory proof-of-concept analysis based on an existing cohort dataset. Complete-case analysis was used due to low missingness, and sex-stratified modeling was given precedence over formal interaction testing. Variables such as alcohol use, liver disease, medication exposure, inflammatory markers, nutritional variables, and comorbidity burden were not available in sufficiently complete form for inclusion, so part of the observed signal for biomarkers such as GGT and albumin may reflect unmeasured clinical contexts rather than direct frailty-specific pathways. The present revision does not yet provide TRIPOD (Transparent Reporting of a Multivariable Prediction Model for Individual Prognosis or Diagnosis)-compliant reporting, optimism-corrected full model validation, or direct benchmarking against simpler baseline models (eg, age-only or age-plus-albumin), all of which should be incorporated in future validation-oriented refinement.

### Broader Implications

Our proof-of-concept analysis supports the viability of this modeling approach, and the next logical step is model refinement. A prospective, multicenter cohort with repeated biomarker assessments and adjudicated clinical outcomes would provide a more appropriate framework to confirm temporality and improve generalizability. External validation in Latin American populations, integration of additional biological data layers, and comparison with machine-learning approaches may further improve predictive performance. Because the proposed models rely on routinely available laboratory parameters, they are particularly suitable for implementation in primary care and resource-limited settings, where they could help prioritize older adults for further geriatric assessment when specialized frailty evaluations are not immediately available. Embedding the frailty-probability equations within electronic health record dashboards or mobile screening tools could enable real-time, low-cost risk stratification in primary care and remote geriatric care. By facilitating earlier identification of risk patterns from routinely available laboratory data, this approach may contribute to more individualized and sex-sensitive frailty management strategies, consistent with the broader goal of precision geriatric care.

Recent discussions in clinical AI emphasize that real-world implementation requires explainability, fairness, human oversight, and careful workflow integration, so computational models should be developed to augment clinicians through earlier detection, smarter triage, and more targeted prevention rather than replace clinical judgment [45]. Likewise, work in adjacent computational health fields reinforces the need for interpretable pipelines, rigorous validation, and multicohort generalizability before routine implementation [46]. Taken together, these considerations position the present work not only as a statistical proof of concept but also as an early translational step toward clinically embedded, digitally supported frailty screening in aging care.

### Conclusions

From routinely available biochemical and hematological data, frailty, operationalized using the FTS-12 score, was modeled with moderate accuracy; sex-specific models demonstrated consistent predictive performance in both male and female participants. The final models included albumin, age, GGT, and a few composite risk functions that are biologically plausible correlates of frailty-related risk. For transparency and future validation, the final equations (albumin in g/dL and GGT in U/L) are given in Multimedia Appendix 1. Currently, these models should not be viewed as nomograms or screening tools with clinically useful biomarker thresholds, but rather as instruments for continuous frailty-risk estimation.

The models offer clinically interpretable signals that could support future workflow-integrated frailty risk stratification, despite the modest explained variance. This strategy may support digitally enabled screening, earlier frailty-oriented assessment, and prioritization for comprehensive geriatric assessment in primary care, geriatric care, and remote care settings following appropriate external and prospective validation. Rather than establishing definitive clinical decision rules, this work was designed to evaluate feasibility, computational stability, and interpretability as a secondary exploratory proof-of-concept analysis based on an existing cohort. Before regular clinical use, additional validation, calibration, and implementation-focused research will be necessary.

## Supplementary material

10.2196/76500 Multimedia Appendix 1 Mathematical equations for the sex-specific multiple and logistic regression models.

10.2196/76500 Multimedia Appendix 2 Statistical rationale for model development, variable selection, bootstrapping, and multicollinearity assessment.

10.2196/76500 Multimedia Appendix 3 Descriptive statistics, selected risk factors, predicted probabilities, and receiver operating characteristic analysis results for the sex-specific regression models.

10.2196/76500 Multimedia Appendix 4 Scatter plots illustrating relationships among composite risk factors, Frailty Trait Scale-12 scores, age, and modeled frailty probabilities in the sex-specific regression analyses.

## Abbreviations

AIC: Akaike information criterion
AUC: area under the curve
CRF: composite risk factor
FI: Frailty Index
FTS: Frailty Trait Scale
GGT: gamma-glutamyl transferase
RDW: red cell distribution width
ROC: receiver operating characteristic
TRIPOD: Transparent Reporting of a Multivariable Prediction Model for Individual Prognosis or Diagnosis
TSHA: Toledo Study for Healthy Aging
VIF: variance inflation factor

## References

[1] Morley JE, Vellas B, van Kan GA (2013). Frailty consensus: a call to action. J Am Med Dir Assoc.

[2] Martin FC, O’Halloran AM (2020). Tools for assessing frailty in older people: general concepts. Adv Exp Med Biol.

[3] Fried LP, Tangen CM, Walston J (2001). Frailty in older adults: evidence for a phenotype. J Gerontol A Biol Sci Med Sci.

[4] Yamada M, Arai H (2023). Understanding social frailty. Arch Gerontol Geriatr.

[5] Verghese J, Ayers E, Sathyan S (2021). Trajectories of frailty in aging: prospective cohort study. PLoS One.

[6] Dibello V, Zupo R, Sardone R (2021). Oral frailty and its determinants in older age: a systematic review. Lancet Healthy Longev.

[7] Di Ciaula A, Portincasa P (2020). The environment as a determinant of successful aging or frailty. Mech Ageing Dev.

[8] Hoogendijk EO, Afilalo J, Ensrud KE, Kowal P, Onder G, Fried LP (2019). Frailty: implications for clinical practice and public health. Lancet.

[9] Cesari M, Prince M, Thiyagarajan JA (2016). Frailty: an emerging public health priority. J Am Med Dir Assoc.

[10] Rockwood K, Mitnitski A (2007). Frailty in relation to the accumulation of deficits. J Gerontol A Biol Sci Med Sci.

[11] García-García FJ, Carcaillon L, Fernandez-Tresguerres J (2014). A new operational definition of frailty: the Frailty Trait Scale. J Am Med Dir Assoc.

[12] Rodríguez-Mañas L, Féart C, Mann G (2013). Searching for an operational definition of frailty: a Delphi method based consensus statement: the frailty operative definition-consensus conference project. J Gerontol A Biol Sci Med Sci.

[13] García-García FJ, Carnicero JA, Losa-Reyna J (2020). Frailty Trait Scale-Short Form: a frailty instrument for clinical practice. J Am Med Dir Assoc.

[14] Alonso-Bouzón C, Carcaillon L, García-García FJ, Amor-Andrés MS, El Assar M, Rodríguez-Mañas L (2014). Association between endothelial dysfunction and frailty: the Toledo Study for Healthy Aging. Age (Dordr).

[15] Rosner B (2015). Fundamentals of Biostatistics.

[16] Forthofer RN, Lee ES, Hernandez M (2006). Biostatistics: A Guide to Design, Analysis and Discovery.

[17] Draper NR, Smith H (1998). Applied Regression Analysis.

[18] Rawlings JO, Pantula SG, Dickey DA (1998). Applied Regression Analysis: A Research Tool.

[19] Searle SR (1997). Linear Models.

[20] Hosmer DW, Lemeshow S, Sturdivant RX (2013). Applied Logistic Regression.

[21] Sepúlveda M, Arauna D, García F, Albala C, Palomo I, Fuentes E (2022). Frailty in aging and the search for the optimal biomarker: a review. Biomedicines.

[22] Zhang L, Yang P, Yin F, Zhang J, Zhao B, Zhou J (2024). Association between frailty and hypoproteinaemia in older patients: meta-analysis and systematic review. BMC Geriatr.

[23] Riviati N, Indrajaya T (2024). Serum albumin as prognostic marker for older adults in hospital and community settings. Gerontol Geriatr Med.

[24] Palmer K, Vetrano DL, Marengoni A (2018). The relationship between anaemia and frailty: a systematic review and meta-analysis of observational studies. J Nutr Health Aging.

[25] Li CM, Chao CT, Chen SI, Han DS, Huang KC (2020). Elevated red cell distribution width is independently associated with a higher frailty risk among 2,932 community-dwelling older adults. Front Med (Lausanne).

[26] Kang MG, Jung HW, Kim BJ (2025). Elevated serum gamma-glutamyl transferase as a risk factor for frailty in older men: a nationwide population-based study. J Cachexia Sarcopenia Muscle.

[27] Gobbens RJJ, van der Ploeg T (2021). The prediction of mortality by quality of life assessed with the WHOQOL-BREF: a longitudinal analysis at the domain and item levels using a seven-year follow-up period. Qual Life Res.

[28] Gobbens RJJ, van Assen M, Augustijn H, Goumans M, van der Ploeg T (2021). Prediction of mortality by the Tilburg Frailty Indicator (TFI). J Am Med Dir Assoc.

[29] van der Ploeg T, Gobbens RJJ, Salem BE (2023). Bayesian techniques in predicting frailty among community-dwelling older adults in the Netherlands. Arch Gerontol Geriatr.

[30] van der Ploeg T, Gobbens R (2022). A comparison of different modeling techniques in predicting mortality with the Tilburg Frailty Indicator: longitudinal study. JMIR Med Inform.

[31] Bencivenga L, Picaro F, Ferrante L (2022). Muscle ultrasound as imaging domain of frailty. Front Med (Lausanne).

[32] Searle SD, Mitnitski A, Gahbauer EA, Gill TM, Rockwood K (2008). A standard procedure for creating a frailty index. BMC Geriatr.

[33] Myers RH, Montgomery DC, Vining GG, Robinson TJ (2010). Generalized Linear Models: With Applications in Engineering and the Sciences.

[34] Vittinghoff E, Glidden DV, Shiboski SC, McCulloch CE (2012). Regression Methods in Biostatistics: Linear, Logistic, Survival, and Repeated Measures Models.

[35] Christensen R (2006). Log-Linear Models and Logistic Regression.

[36] Wang C, Fang X, Tang Z (2022). A frailty index based on routine laboratory data predicts increased risk of mortality in Chinese community-dwelling adults aged over 55 years: a five-year prospective study. BMC Geriatr.

[37] Pasternak E, Freud T, Press Y (2025). Association between the frailty index based on laboratory tests and all-cause mortality in hospitalized older adults: retrospective cohort study. JMIR Aging.

[38] Liu H, Jiao J, Zhu M (2021). An early predictive model of frailty for older inpatients according to nutritional risk: protocol for a cohort study in China. BMC Geriatr.

[39] Álvarez-Sánchez N, Álvarez-Ríos AI, Guerrero JM (2020). Homocysteine and C-reactive protein levels are associated with frailty in older Spaniards: the Toledo Study for Healthy Aging. J Gerontol A Biol Sci Med Sci.

[40] Lee S, Song D, Shin S, Hong N, Rhee Y (2022). Elevated serum γ-glutamyl transferase is associated with low muscle function in adults independent of muscle mass. Nutrition.

[41] Kim KJ, Hong N, Yu MH (2022). Elevated gamma-glutamyl transpeptidase level is associated with an increased risk of hip fracture in postmenopausal women. Sci Rep.

[42] Arauna D, Navarrete S, Albala C (2024). Understanding the role of oxidative stress in platelet alterations and thrombosis risk among frail older adults. Biomedicines.

[43] Gbur EE, Stroup WW, McCarter KS (2012). Analysis of Generalized Linear Mixed Models in the Agricultural and Natural Resources Sciences.

[44] Puukka K, Hietala J, Koivisto H, Anttila P, Bloigu R, Niemelä O (2006). Age-related changes on serum ggt activity and the assessment of ethanol intake. Alcohol Alcohol.

[45] Ogut E (2025). Artificial intelligence in clinical medicine: challenges across diagnostic imaging, clinical decision support, surgery, pathology, and drug discovery. Clin Pract.

[46] Ogut E (2025). Graph-theoretical mapping of cortical and subcortical network alterations in preclinical neurodegeneration. Discov Neurosci.

