# Supplementary material for: Modeling Frailty Risk Based on Biochemical and Hematological Parameters Using Multiple and Logistic Regression: Cross-Sectional Analysis of the Toledo Study for Healthy Aging
Source: Interact J Med Res. 2026 Oct 30;15:e76500. doi: 10.2196/76500 (PMC13722519; doi:10.2196/76500)
Supplement: Supplementary file 2 [file ijmr-v15-e76500-s002.docx]

***The regression equation for predicting the frailty value***

The analytic expression of a predictive function within the context of multiple regression is as follows:

(S1)

where, $b_{0}$is the intercept parameter, while the coefficients $b_{1},\text{ }b_{2},\text{ }b_{3},...$ are the parameters to be estimated from the data, and$X_{1},\text{ }X_{2},\text{ }X_{3},\text{ }\ldots$. are the predictive (independent) variables. Depending on the context, these variables are referred to as independent variables, risk factors, predictors, or regressors.

***The theory behind the logistic regression***

We defined a binary response variable, $Y$, such that $Y=1$ if an individual’s FTS-12 score exceeded the third quartile (i.e., fell within the highest – fourth - quarter), and $Y=0$ otherwise. This can be mathematically expressed as:

$\begin{matrix} Y=1\text{ }if\text{ }FTS12\text{ }\geq\text{ }55 \\ Y=0\text{ }if\text{ }FTS12\text{ < }55 \end{matrix}$ (S2)

In the context of the logistic regression, we defined $p_{j}$as “*the probability that the frailty score of the j-th individual exceeded the third quartile of FTS-12 values*” and $1-p_{j}$as “*the probability that the frailty score of the j-th individual does not exceeded the third quartile of FTS-12 values*.” Consequently, the odds that an individual's frailty score exceeded the third quartile of FTS-12 values is given by the “odds” $p_{j}/\left(1-p_{j}\right).$

We assumed that the response variable $Y_{j}$ is a “Bernoulli random variable” with the following probability distribution [21]:

| $Y_{j}$ | Probability |
| --- | --- |
| 1 |  |
| 0 | $P\left(Y_{j}=0\right)=1-p_{j}$ |

The statistical properties of the response variable $Y_{j}$ can significantly impact the choice of a linear predictive response function [21]. When the response variable is binary, empirical evidence suggests that a nonlinear response function is more appropriate. This function, known as the “*logistic response function*” [21], employs the “*logit*” transformation to achieve linearization. Therefore, the logistic response function used to model the odds of an FTS-12 value falling in the fourth quartile is as follows:

$LR=Log-likelihood\text{ }ratio=Ln\left(\frac{p}{1-p}\right)=b_{0}+b_{1}X_{1}+b_{2}X_{2}+b_{3}X_{3}+\cdots$ (S3)

The natural logarithm of the odds (l*ogit* transformation) is referred to as the “*log likelihood ratio*” (LR) or “*log-odds*,” depending on the context. The linearized logistic response function allows for modeling the probability that the FTS-12 for any individual falls within the fourth quarter $\left(p_{j}\right)$, rather than directly modeling the binary response variable $\left(Y_{j}\right)$. The fundamental assumption here is that the likelihood ratio of the outcome (falling or not) is linearly related to the predictive independent variables $X_{1},X_{2},X_{3},\ldots$. Additionally, the intercept parameter in the function is denoted as $b_{0}$, while $b_{1},\text{ }b_{2},\text{ }b_{3},...$ represent the parameters in the predictive function to be estimated from the data. Depending on the context, the selected variables may also be referred to as “risk factors” or “risk covariates.” Two linear predictive functions for the likelihood ratio were also modeled, following the same criteria used in the multiple regression analysis described above. Separate predictive equations for the likelihood ratio were modeled for each sex. Regression analysis was conducted using JMP 13.2 software, along with the LOGISTIC procedure in SAS 9.4 and SAS Enterprise Guide 7.1. A stepwise regression approach, as outlined by Hosmer and Lemeshow (2013), was applied as the selection method. After selecting the function, we used SAS to obtain the predicted value for each *j-th* individual in the dataset. This was done using

$XBeta_{j}=b_{0}+b_{1}X_{1j}+b_{2}X_{2j}+b_{3}X_{3j}+\cdots$ (S4)

Subsequently, we calculated the odds for each *j-th* individual

 (S5)

Finally, we estimated the probability that the frailty FTS-12 value for each *j-th* individual falls within the fourth quarter using the following expression:

 (S6)

We further estimated the “*odds ratio*” $\left(OR_{i}\right)$associated with each risk factor $(X_{i})$. Let and represent the probabilities that frailty is larger than the third quartile when the *i-th* risk factor$X_{i}$ takes values of 1 and 0, respectively. Therefore, the “*odds ratio*” associated with the risk factor $X_{i}$was estimated as follows:

$\begin{matrix} \hat{O}R_{i}=Ln\left(\frac{P\left(X_{i}=1\right)/\left[1-P\left(X_{i}=1\right)\right]}{P\left(X_{i}=0\right)/\left[1-P\left(X_{i}=0\right)\right]}\right)=\hat{\beta}_{i}=b_{i} \\ \hat{O}R_{i}=\frac{P\left(X_{i}=1\right)/\left[1-P\left(X_{i}=1\right)\right]}{P\left(X_{i}=0\right)/\left[1-P\left(X_{i}=0\right)\right]}=\exp\left(\hat{\beta}_{i}\right)=\exp\left(b_{i}\right) \end{matrix}$ (S7)

where $b_{i}$is the estimate of the regression parameter associated to risk factor $X_{i}$[21].

***Main results: The selected model equations***

The multiple regression equation chosen for the women’s data is as follows:

The multiple regression equation derived for the men’s dataset is:

The logistic regression equation chosen for the women’s data is as follows:

The logistic regression equation selected for the men’s data is as follows:

***Bibliography***

Hosmer D, Lemeshow S, Sturdivant RX. Applied Logistic Regression. 3^rd^ edition. NJ. John Wiley & Sons. 2013.

***Summary of Collinearity Assessment in Multiple Regression Analysis***

**Table S1. Multicollinearity Analysis. Multiple Regression Analysis. Female Data. Variable FTS-12**

**Table S2. Multicollinearity Analysis. Multiple Regression Analysis. Male Data. Variable FTS-12**

***Summary of Collinearity Assessment in Logistic Regression Analysis***

**Table S3. Multicollinearity Analysis. Logistic Regression Analysis. Female Data**

**Table S4. Multicollinearity Analysis. Logistic Regression Analysis. Male Data**

***Summary of Bootstrapping Analysis in Multiple Regression Analysis.***

***Female Data***

Intercept

Granulocytes (%)

Urea (mg/dL)

Uric Acid (mg/dL)

Creatinine (mg/dL)

Bilirubin Total (mg/dL)

Triglycerides (mg/dL)

Chlorine (mEq/L)

GGT (U/L)

$$\frac{Ln\left(Alkaline\text{ }Phosphatase\times Age\right)}{Albumin}$$

R-squared

***Summary of Bootstrapping Analysis in Multiple Regression Analysis.***

***Male Data***

Intercept

Leukocites (x10^3^/µL)

Eosinophil (%)

Uric Acid (mg/dL)

Triglycerides (mg/dL)

Phosphorus (mg/dL)

Chlorine (mEq/L)

GGT (U/L)

$$\frac{Ln\left(Albumin\right)\times100}{Age}$$

$$\frac{Ln\left(Alkaline\text{ }Phosphatase\times Age\right)}{Albumin}$$

R-squared
