# Supplementary material for: Modeling Frailty Risk Based on Biochemical and Hematological Parameters Using Multiple and Logistic Regression: Cross-Sectional Analysis of the Toledo Study for Healthy Aging
Source: Interact J Med Res. 2026 Oct 30;15:e76500. doi: 10.2196/76500 (PMC13722519; doi:10.2196/76500)
Supplement: Supplementary file 3 [file ijmr-v15-e76500-s003.docx]

Supplementary tables presenting descriptive statistics, selected risk factors, predicted probabilities, and ROC analysis results for the sex-specific multiple and logistic regression models.

**Table S1**. Descriptive Statistics and Characteristics of the Dependent Variable and Risk Factors in the Selected Model: Women’s Data.

| **Variable**  **(units)** | **Mean** | **Standard Deviation** | **n** | **Minimum**  **Value** | **Maximum**  **Value** | **Q1** | **Q2=Me** | **Q3** | **CV** |
| --- | --- | --- | --- | --- | --- | --- | --- | --- | --- |
| FTS12 | 46.68 | 15.99 | 847 | 6.00 | 92.11 | 34.78 | 46.00 | 58.00 | 34.26 |
| Age (years) | 75.04 | 5.84 | 847 | 65.00 | 95.00 | 71.00 | 75.00 | 78.00 | 7.78 |
| Granulocytes (%) | 56.23 | 8.88 | 838 | 30.80 | 81.10 | 50.10 | 56.10 | 62.10 | 15.79 |
| Urea (mg/dL) | 44.09 | 11.51 | 829 | 19.00 | 81.00 | 36.00 | 43.00 | 50.00 | 26.10 |
| Uric Acid (mg/dL) | 4.81 | 1.29 | 840 | 1.40 | 8.90 | 3.85 | 4.70 | 5.60 | 26.93 |
| Creatinine (mg/dL) | 0.76 | 0.20 | 840 | 0.20 | 1.50 | 0.60 | 0.70 | 0.90 | 25.82 |
| Bilirubin (mg/dL) | 0.55 | 0.24 | 839 | 0.01 | 1.50 | 0.40 | 0.50 | 0.69 | 44.41 |
| Triglycerides (mg/dL) | 112.00 | 45.72 | 823 | 30.00 | 265.00 | 79.00 | 102.00 | 140.00 | 40.82 |
| Chlorine (mEq/L) | 102.80 | 2.99 | 842 | 93.00 | 112.00 | 101.00 | 103.00 | 105.00 | 2.91 |
| GGT (U/L) | 22.47 | 12.95 | 829 | 4.00 | 90.00 | 14.00 | 19.00 | 26.00 | 57.61 |
| Ln(RDWxAge3/Alb) | 14.12 | 0.28 | 825 | 13.58 | 15.14 | 13.92 | 14.09 | 14.30 | 1.95 |
| Ln(AlkPhosxAge)/Alb | 2.03 | 0.15 | 829 | 1.71 | 2.63 | 1.93 | 2.02 | 2.12 | 7.35 |
| RDW (%) | 13.80 | 1.05 | 828 | 11.60 | 18.50 | 13.10 | 13.60 | 14.20 | 7.64 |
| Albumin (g/dL) | 4.27 | 0.25 | 843 | 3.40 | 4.90 | 4.10 | 4.30 | 4.40 | 5.87 |
| Alkaline Phosphatase (U/L) | 79.77 | 22.98 | 842 | 28.00 | 171.00 | 63.00 | 76.00 | 93.00 | 28.81 |

**Legend**: Ln(RDWxAge3/Alb)=and Ln(AlkPhosxAge)/Alb=$\frac{Ln\left(Alkaline\text{ }Phosphatase\times Age\right)}{Albumin}$. Q1: First (lower) quartile; Q2: Second (Median) quartil; Q3: Third (Upper) quartil.

**Table S2.** Descriptive Statistics and Characteristics of the Dependent Variable and Risk Factors in the Selected Model: Men’s Data.

| **Variable**  **(units)** | **Mean** | **Standard Deviation** | **n** | **Minimum**  **Value** | **Maximum**  **Value** | **Q1** | **Q2=Me** | **Q3** | **CV** |
| --- | --- | --- | --- | --- | --- | --- | --- | --- | --- |
| FTS12 | 39.62 | 15.43 | 637 | 0.00 | 84.00 | 28.00 | 38.00 | 50.00 | 38.94 |
| Age (years) | 74.95 | 5.63 | 637 | 65.00 | 99.00 | 71.00 | 75.00 | 78.00 | 7.51 |
| Leukocites (x10^3^/µL) | 7.06 | 1.66 | 626 | 3.20 | 12.60 | 5.90 | 6.90 | 8.10 | 23.47 |
| Eosinophil (%) | 3.16 | 1.78 | 623 | 0.00 | 9.40 | 1.90 | 2.70 | 4.00 | 56.30 |
| Uric Acid (mg/dL) | 5.61 | 1.35 | 625 | 2.60 | 9.50 | 4.60 | 5.50 | 6.40 | 24.04 |
| Phosphorus (mg/dL) | 3.07 | 0.49 | 626 | 1.80 | 4.90 | 2.70 | 3.00 | 3.40 | 15.92 |
| Clhorine (mEq/L) | 102.67 | 3.05 | 629 | 94.00 | 113.00 | 101.00 | 103.00 | 105.00 | 2.97 |
| GGT (U/L) | 28.58 | 16.49 | 620 | 8.00 | 103.00 | 17.00 | 24.00 | 35.00 | 57.68 |
| Ln(Alb)x100/Age | 1.97 | 0.19 | 631 | 1.36 | 2.41 | 1.83 | 1.98 | 2.09 | 9.56 |
| Ln(AlkPhosxAge)/Alb | 1.99 | 0.16 | 625 | 1.53 | 2.76 | 1.89 | 1.97 | 2.07 | 7.81 |
| Triglycerides | 104.48 | 43.18 | 617 | 32 | 271 | 73 | 96 | 127 | 41.32 |
| Albumin (g/dL) | 4.35 | 0.27 | 631 | 3.30 | 5.00 | 4.20 | 4.40 | 4.50 | 6.19 |
| Alkaline Phosphatase  (U/L) | 75.31 | 21.82 | 620 | 17.00 | 174.00 | 59.50 | 72.00 | 87.00 | 28.97 |

**Legend**: Ln(Alb)x100/Age=$\frac{Ln\left(Albumin\right)\times100}{Age}$and Ln(AlkPhosxAge)/Alb=$\frac{Ln\left(Alkaline\text{ }Phosphatase\times Age\right)}{Albumin}$. Q1: First (lower) quartile; Q2: Second (Median) quartile; Q3: Third (Upper) quartile.

**Table S3**. Relevant statistics of predicted probabilities and risk factors in the selected logistic regression Model for women’s data.

| **Variable** | **Mean** | **Standard Deviation** | **n** | **Minimum**  **Value** | **Maximum**  **Value** | **Q1** | **Q2=Me** | **Q3** | **CV** |
| --- | --- | --- | --- | --- | --- | --- | --- | --- | --- |
| Predicted Probability | 0.27 | 0.22 | 735 | 0.01 | 0.97 | 0.10 | 0.20 | 0.39 | 80 |
| FTS12 | 46.68 | 15.99 | 847 | 6.00 | 92.11 | 34.78 | 46.00 | 58.00 | 34.26 |
| Age (years) | 75.04 | 5.84 | 847 | 65.00 | 95.00 | 71.00 | 75.00 | 78.00 | 7.78 |
| Granulocytes (%) | 56.23 | 8.88 | 838 | 30.80 | 81.10 | 50.10 | 56.10 | 62.10 | 15.79 |
| Urea (mg/dL) | 44.09 | 11.51 | 829 | 19.00 | 81.00 | 36.00 | 43.00 | 50.00 | 26.10 |
| Albumin (g/dL) | 4.27 | 0.25 | 843 | 3.40 | 4.90 | 4.10 | 4.30 | 4.40 | 5.87 |
| GGT (U/L) | 22.47 | 12.95 | 829 | 4.00 | 90.00 | 14.00 | 19.00 | 26.00 | 57.61 |
| Alkaline Phosphate (U/L) | 79.77 | 22.98 | 842 | 28.00 | 171.00 | 63.00 | 76.00 | 93.00 | 28.81 |
| LDL (mg/dL) | 119.54 | 30.78 | 775 | 40.00 | 219.00 | 98.00 | 119.00 | 140.00 | 25.75 |
| Ln(Alb)x100/Age | 1.97 | 0.19 | 632 | 1.36 | 2.41 | 1.83 | 1.98 | 2.07 | 9.39 |

**Legend**: Ln(Alb)x100/Age=$\frac{Ln\left(Albumin\right)\times100}{Age}$. Q1: First (lower) quartile; Q2: Second (Median) quartile; Q3: Third (Upper) quartile; CV: coefficient of variation.

**Table S4**. Relevant statistics of predicted probabilities and risk factors in the selected logistic regression Model for men’s data.

| **Variable** | **Mean** | **Standard Deviation** | **n** | **Minimum**  **Value** | **Maximum**  **Value** | **Q1** | **Q2=Me** | **Q3** | **CV** |
| --- | --- | --- | --- | --- | --- | --- | --- | --- | --- |
| Predicted probability | 0.18 | 0.185 | 564 | 0.001 | 0.85 | 0.041 | 0.108 | 0.26 | 104.55 |
| FTS12 | 39.62 | 15.43 | 637 | 0.00 | 84.00 | 28.00 | 38.00 | 50.00 | 38.94 |
| Age (years) | 74.95 | 5.63 | 637 | 65.00 | 99.00 | 71.00 | 75.00 | 78.00 | 7.51 |
| Albumin (g/dL) | 4.35 | 0.27 | 631 | 3.30 | 5.00 | 4.20 | 4.40 | 4.50 | 6.19 |
| Phosphorus (mg/dL) | 3.07 | 0.49 | 626 | 1.80 | 4.90 | 2.70 | 3.00 | 3.40 | 15.92 |
| Alkaline Phosphate (U/L) | 75.31 | 21.82 | 628 | 17.00 | 174.00 | 59.50 | 72.00 | 87.00 | 28.97 |
| Triglycerides (mg/dL) | 104.48 | 43.18 | 617 | 32 | 271 | 73 | 96 | 127 | 41.32 |
| Hemoglobin (g/dL) | 14.90 | 1.45 | 630 | 9.60 | 18.80 | 14.10 | 15.10 | 15.80 | 9.71 |
| CHCM (g/L) | 33.62 | 0.90 | 610 | 30.50 | 36.70 | 33.10 | 33.60 | 34.20 | 2.68 |
| Lactate Dehydrogenase (U/L) | 350.73 | 57.90 | 622 | 163.00 | 546.00 | 313.00 | 345.00 | 387.00 | 16.51 |
| Hemox10E5/EAge3 | 3.67 | 0.92 | 630 | 1.41 | 6.19 | 3.04 | 3.60 | 4.29 | 25.11 |

**Legend**: Hemox10E5/Age3=. Q1: First (lower) quartile; Q2: Second (Median) quartile; Q3: Third (Upper) quartile; CV: coefficient of variation.

**Table S5**. Associated statistics for the ROC analysis of selected logistic regression equation for Women’s data and selected Model.

| **ROC Associated Statistics** | | | | | | | | | |
| --- | --- | --- | --- | --- | --- | --- | --- | --- | --- |
| **Variable in the model (Risk Factor)** |  | **Mann-Whitney** | | | |  | **Somers' D** | **Gamma** | **Tau-a** |
|  |  | **AUC** | **Standard Error** | **95% Wald Confidence Limits** | |  |  |  |  |
| All variables (risk factors) included in the model |  | 0.7987 | 0.0181 | 0.7632 | 0.8341 |  | 0.5974 | 0.5974 | 0.2370 |
| Ln(Alb)x100/Age |  | 0.7646 | 0.0197 | 0.7260 | 0.8031 |  | 0.5291 | 0.5318 | 0.2099 |
| Granulocytes (%) |  | 0.6201 | 0.0237 | 0.5736 | 0.6665 |  | 0.2402 | 0.2409 | 0.0953 |
| Alkaline Phosphatase (U/L) |  | 0.6046 | 0.0233 | 0.5589 | 0.6502 |  | 0.2091 | 0.2119 | 0.0830 |
| GGT (U/L) |  | 0.5474 | 0.0242 | 0.5000 | 0.5948 |  | 0.0948 | 0.0981 | 0.0376 |
| LDL (mg/dL) |  | 0.5677 | 0.0242 | 0.5202 | 0.6151 |  | 0.1353 | 0.1365 | 0.0537 |
| Urea (mg/dL) |  | 0.5676 | 0.0244 | 0.5197 | 0.6156 |  | 0.1353 | 0.1388 | 0.0537 |

**Legend**: Ln(Alb)x100/Age=$\frac{Ln\left(Albumin\right)\times100}{Age}$. AUC: Area Under the Curve

**Table S6**. Associated statistics for the ROC analysis of selected logistic regression equation for Men’s data and selected Model.

| **ROC Associated Statistics** | | | | | | | | | |
| --- | --- | --- | --- | --- | --- | --- | --- | --- | --- |
| **Variable in the model**  **(Risk Factor)** |  | **Mann-Whitney** | | | |  | **Somers' D** | **Gamma** | **Tau-a** |
|  |  | **AUC** | **Standard Error** | **95% Wald**  **Confidence Limits** | |  |  |  |  |
| All variables (risk factors) included in the model |  | 0.8238 | 0.0201 | 0.7844 | 0.8632 |  | 0.6475 | 0.6475 | 0.1892 |
| Hemox10E5/Ed3 |  | 0.7354 | 0.0255 | 0.6853 | 0.7855 |  | 0.4708 | 0.4713 | 0.1376 |
| Albumin (g/dL) |  | 0.7126 | 0.0276 | 0.6586 | 0.7666 |  | 0.4252 | 0.4717 | 0.1243 |
| Alkaline Phosphatase (U/L) |  | 0.6018 | 0.0309 | 0.5413 | 0.6623 |  | 0.2036 | 0.2064 | 0.0595 |
| Triglycerides (mg/dL) |  | 0.5751 | 0.0326 | 0.5112 | 0.6389 |  | 0.1501 | 0.1512 | 0.0439 |
| CHCM (g/L) |  | 0.5669 | 0.0310 | 0.5061 | 0.6277 |  | 0.1338 | 0.1389 | 0.0391 |
| Phosphorus (mg/dL) |  | 0.5495 | 0.0332 | 0.4845 | 0.6145 |  | 0.0990 | 0.1047 | 0.0289 |
| Lactate Dehydrogenase (U/L) |  | 0.5066 | 0.0335 | 0.4410 | 0.5722 |  | 0.0131 | 0.0132 | 0.00384 |

**Legend:** Hemox10E5/Ed3=. AUC: Area Under the Curve
