# Supplementary material for: Modeling Frailty Risk Based on Biochemical and Hematological Parameters Using Multiple and Logistic Regression: Cross-Sectional Analysis of the Toledo Study for Healthy Aging
Source: Interact J Med Res. 2026 Oct 30;15:e76500. doi: 10.2196/76500 (PMC13722519; doi:10.2196/76500)
Supplement: Supplementary file 4 [file ijmr-v15-e76500-s004.docx]

The multiple regression equation chosen for the women’s data is as follows:

The multiple regression equation derived for the men’s dataset is:

The logistic regression equation chosen for the women’s data is as follows:

The logistic regression equation selected for the men’s data is as follows:
